# The oocyte microenvironment is altered in adolescents compared to oocyte donors

**DOI:** 10.1093/hropen/hoae047

**Published:** 2024-08-06

**Authors:** Dilan Gokyer, Sophia Akinboro, Luhan T Zhou, Anna Kleinhans, Monica M Laronda, Francesca E Duncan, Joan K Riley, Kara N Goldman, Elnur Babayev

**Affiliations:** Department of Obstetrics and Gynecology, Feinberg School of Medicine, Northwestern University, Chicago, IL, USA; Department of Obstetrics and Gynecology, Feinberg School of Medicine, Northwestern University, Chicago, IL, USA; Department of Neuroscience, Weinberg College of Arts and Sciences, Northwestern University, Evanston, IL, USA; Department of Obstetrics and Gynecology, Feinberg School of Medicine, Northwestern University, Chicago, IL, USA; Department of Obstetrics and Gynecology, Northwestern Medicine Center for Fertility and Reproductive Medicine, Chicago, IL, USA; Department of Obstetrics and Gynecology, Feinberg School of Medicine, Northwestern University, Chicago, IL, USA; Department of Pediatrics, Feinberg School of Medicine, Northwestern University, Chicago, IL, USA; Basic and Preclinical Science, Stanley Manne Children’s Research Institute, Ann & Robert H. Lurie Children’s Hospital of Chicago, Chicago, IL, USA; Department of Obstetrics and Gynecology, Feinberg School of Medicine, Northwestern University, Chicago, IL, USA; Department of Obstetrics and Gynecology, Feinberg School of Medicine, Northwestern University, Chicago, IL, USA; Department of Obstetrics and Gynecology, Northwestern Medicine Center for Fertility and Reproductive Medicine, Chicago, IL, USA; Department of Obstetrics and Gynecology, Feinberg School of Medicine, Northwestern University, Chicago, IL, USA; Department of Obstetrics and Gynecology, Northwestern Medicine Center for Fertility and Reproductive Medicine, Chicago, IL, USA; Department of Obstetrics and Gynecology, Feinberg School of Medicine, Northwestern University, Chicago, IL, USA; Department of Obstetrics and Gynecology, Northwestern Medicine Center for Fertility and Reproductive Medicine, Chicago, IL, USA

**Keywords:** cumulus cells, follicular fluid, fertility preservation, egg quality, children, RNA-seq, cytokine analysis

## Abstract

**STUDY QUESTION:**

Do the molecular signatures of cumulus cells (CCs) and follicular fluid (FF) of adolescents undergoing fertility preservation differ from that of oocyte donors?

**SUMMARY ANSWER:**

The microenvironment immediately surrounding the oocyte, including the CCs and FF, is altered in adolescents undergoing fertility preservation compared to oocyte donors.

**WHAT IS KNOWN ALREADY:**

Adolescents experience a period of subfecundity following menarche. Recent evidence suggests that this may be at least partially due to increased oocyte aneuploidy. Reproductive juvenescence in mammals is associated with suboptimal oocyte quality.

**STUDY DESIGN, SIZE, DURATION:**

This was a prospective cohort study. Adolescents (10–19 years old, n = 23) and oocyte donors (22–30 years old, n = 31) undergoing ovarian stimulation and oocyte retrieval at a single center between 1 November 2020 and 1 May 2023 were enrolled in this study.

**PARTICIPANTS/MATERIALS, SETTING, METHODS:**

Patient demographics, ovarian stimulation, and oocyte retrieval outcomes were collected for all participants. The transcriptome of CCs associated with mature oocytes was compared between adolescents (10–19 years old, n = 19) and oocyte donors (22–30 years old, n = 19) using bulk RNA-sequencing. FF cytokine profiles (10–19 years old, n = 18 vs 25–30 years old, n = 16) were compared using cytokine arrays.

**MAIN RESULTS AND THE ROLE OF CHANCE:**

RNA-seq analysis revealed 581 differentially expressed genes in CCs of adolescents relative to oocyte donors, with 361 genes downregulated and 220 upregulated. Genes enriched in pathways involved in cell cycle and cell division (e.g. GO: 1903047, *P* = 3.5 × 10^−43^; GO: 0051983, *P* = 4.1 × 10^−30^; GO: 0000281, *P* = 7.7 × 10^−15^; GO: 0044839, *P* = 5.3 × 10^−13^) were significantly downregulated, while genes enriched in several pathways involved in cellular and vesicle organization (e.g. GO: 0010256, *P* = 1.2 × 10^−8^; GO: 0051129, *P* = 6.8 × 10^−7^; GO: 0016050, *P* = 7.4 × 10^−7^; GO: 0051640, *P* = 8.1 × 10^−7^) were upregulated in CCs of adolescents compared to oocyte donors. The levels of nine cytokines were significantly increased in FF of adolescents compared to oocyte donors: IL-1 alpha (2-fold), IL-1 beta (1.7-fold), I-309 (2-fold), IL-15 (1.6-fold), TARC (1.9-fold), TPO (2.1-fold), IGFBP-4 (2-fold), IL-12-p40 (1.7-fold), and ENA-78 (1.4-fold). Interestingly, seven of these cytokines have known pro-inflammatory roles. Importantly, neither the CC transcriptomes nor FF cytokine profiles were different in adolescents with or without cancer.

**LARGE SCALE DATA:**

Original high-throughput sequencing data have been deposited in Gene Expression Omnibus (GEO) database with the accession number GSE265995.

**LIMITATIONS, REASONS FOR CAUTION:**

This study aims to gain insights into the associated gamete quality by studying the immediate oocyte microenvironment. The direct study of oocytes is more challenging due to sample scarcity, as they are cryopreserved for future use, but would provide a more accurate assessment of oocyte reproductive potential.

**WIDER IMPLICATIONS OF THE FINDINGS:**

Our findings have implications for the adolescent fertility preservation cycles. Understanding the expected quality of cryopreserved eggs in this age group will lead to better counseling of these patients about their reproductive potential and may help to determine the number of eggs that is recommended to be banked to achieve a reasonable chance of future live birth(s).

**STUDY FUNDING/COMPETING INTEREST(S):**

This project was supported by Friends of Prentice organization SP0061324 (M.M.L. and E.B.), Gesualdo Family Foundation (Research Scholar: M.M.L.), and NIH/NICHD K12 HD050121 (E.B.). The authors have declared that no conflict of interest exists.

WHAT DOES THIS MEAN FOR PATIENTS? After the onset of menstruation, adolescents typically go through a phase where their fertility potential is suboptimal. Recent studies suggest that this may be attributed in part to chromosomal abnormalities which lead to decreased egg quality in adolescents. With an increasing number of adolescents and young adults freezing their eggs for fertility preservation, especially in the context of cancer or expected longstanding use of hormones for gender dysphoria, concerns arise regarding the quality of these frozen eggs. It is essential to understand the reproductive potential of these eggs so that clinicians can provide appropriate guidance to patients regarding their future fertility prospects, and health outcomes for their future children. Our study aimed to evaluate the molecular signature of the immediate oocyte microenvironment which is typically reflective of the quality of the associated egg. We found that this microenvironment is significantly different in adolescents compared to young women with good reproductive potential who donate their eggs to the infertile patients. This study paves the way for an understanding of egg quality for very young patients and has implications for fertility preservation in this age group.

## Introduction

Long-term survival of children with cancers has significantly improved in the last few decades due to advancements in oncology (Armstrong *et al.*, 2016). However, many of the life-saving treatments are toxic to the gonads (Goldman *et al.*, 2017). Patients with cancer report that fertility concerns cause significant distress to them and their family members, and many express a strong desire to preserve the possibility of having a biological child in the future (Mulder *et al.*, 2021). Similarly, patients with gender dysphoria prefer fertility preservation in some cases prior to gender-affirming treatments due to unknown long-term effects of cross-sex hormone therapy on the quality of gametes or to prevent the detrimental psychological impacts of coming off of gender-affirming hormones later in life (Chen and Simons, 2018; Moravek and Obedin-Maliver, 2021). Gamete, embryo, and ovarian/testis tissue cryopreservation are the available options to preserve fertility (ASRM Committee Opinion, 2019). Oocyte cryopreservation following controlled ovarian stimulation is the preferred method for fertility preservation for post-pubertal adolescents (ASRM Committee Opinion, 2021). Adolescence is defined as the phase of life between childhood and adulthood, from ages 10 to 19 (WHO, 2024).

Oocyte quantity and quality are highly dependent on age. With advanced reproductive age, there is a well-documented decrease in gamete quality due to oocyte aneuploidy and mitochondrial dysfunction as well as ovarian stromal inflammation and fibrosis (Wang *et al.*, 2011; Duncan *et al.*, 2012; Briley *et al.*, 2016; Gruhn *et al.*, 2019; Amargant *et al.*, 2020; Beverley *et al.*, 2021; Liu and Gao, 2023). Accumulating evidence also suggests that egg quality may be compromised at the other end of the age spectrum in very young individuals. The period of adolescent sterility or subfecundity is well documented in ethnology studies conducted in native island populations and in isolated communities in India and Thailand. In these communities, lower pregnancy rates were observed among young girls compared to adult women despite regular sexual intercourse in the absence of reliable contraception (Hartman, 1931; Ashley-Montagu, 1939; Wood and Milligan, 1989; Homan *et al.*, 2007; Duncan, 2017). Similarly, a study of 42 493 parous, monogamously married, 19th century women in the Utah Population Database reported that the natural fertility pattern in humans is represented by an inverse U-shaped curve where both young females (15 to early 20 s) and women of advanced reproductive age (mid-30s and above) tend to experience lower fertility rates (Hawkes and Smith, 2010).

Decreased egg quality among very young mammals appears to be phylogenetically conserved (Duncan, 2017). Studies in mice, pig, and non-human primate models demonstrate increased aneuploidy and/or decreased pregnancy rates in juvenescent animals (Mirskaia and Crew, 1931; Koenig and Stormshak, 1993; Wallen and Zehr, 2004; Lechniak *et al.*, 2007; Kusuhara *et al.*, 2021). Studies in humans also raise concerns about relatively poor gamete quality in children and young adults. A review of 15 169 trophectoderm biopsies demonstrates higher aneuploidy rates in patients in their early 20 s (∼40% in women 22–23 years old) compared to patients in their middle to late 20 s (∼20–27% in women 26–30 years old) (Franasiak *et al.*, 2014). Similarly, a recent study of oocytes from women undergoing ovarian tissue cryopreservation (9–39 years old) or oocytes and embryos from women undergoing IVF (20–43 years old) suggests a J-shaped curve representing aneuploidy in humans, with increasing aneuploidy rates as age decreases below 27 years old (Gruhn *et al.*, 2019).

Given the increasing number of fertility preservation cycles in children, adolescents, and young adults, and concerns related to the suboptimal gamete quality in this age group, it is important to understand the quality of the cryopreserved gametes to counsel these patients on their reproductive potential, expected pregnancy outcomes, future offspring health, and to design preventive and/or therapeutic strategies. Following ovarian stimulation, retrieved oocytes are vitrified for future use. However, the immediate microenvironment that surrounds the oocyte, which includes the cumulus cells (CCs) and follicular fluid (FF), can be readily sampled in IVF cycles. Importantly, these cells and biofluids reflect the quality of the associated gamete and key changes that occur with advanced reproductive age (Babayev and Duncan, 2022). CCs support the growth and development of the oocyte, are essential for fertility (Davis *et al.*, 1999; Hizaki *et al.*, 1999; Zhuo *et al.*, 2001; Varani *et al.*, 2002; Fülöp *et al.*, 2003; Salustri *et al.*, 2004), and undergo age-related genomic, transcriptomic, epigenomic, metabolomic, and proteomic changes (Lee *et al.*, 2010; Tsai *et al.*, 2010; McReynolds *et al.*, 2012; Al-Edani *et al.*, 2014; Molinari *et al.*, 2016; Olsen *et al.*, 2020; Babayev and Duncan, 2022). Similarly, FF reflects the metabolism, synthetic capacity, and inflammatory signature of the surrounding granulosa and CCs with advancing age (Adiga *et al.*, 2002; Diez-Fraile *et al.*, 2014; Machlin *et al.*, 2021; Babayev and Duncan, 2022).

In this study, we tested the hypothesis that the biological profile of the immediate microenvironment of the oocyte changes as individuals transition from puberty to reproductive adulthood. To this end, we collected CCs, isolated from mature oocytes, and FF from adolescents undergoing fertility preservation and oocyte donors. We then compared the genome-wide CC transcriptomic signatures and the FF cytokine profile in these populations. To our knowledge, our adolescent group is the largest cohort to date with molecular analysis of the oocyte microenvironment at very young ages. We chose oocyte donors as an older comparison group, because among the patients undergoing oocyte retrieval, they are a select group of presumably fertile adults with good ovarian reserve that represent a population with optimal egg quality. The observed alterations in the immediate oocyte microenvironment of adolescents, including dysregulated biological pathways in CCs and more pro-inflammatory cytokine signature in FF, may be reflective of the underlying differences in gamete quality between these populations. These findings pave the way for our understanding of the reproductive potential of the associated gametes in adolescents.

## Materials and methods

### Population

Adolescent patients (10–19 years old, n = 23) and oocyte donors (22–30 years old, n = 31) undergoing ovarian stimulation and oocyte retrieval at the Northwestern Fertility and Reproductive Medicine Center between 1 November 2020 and 1 May 2023 were enrolled in this study. There were no exclusion criteria for these participants. Samples were collected from a single ovarian stimulation cycle for each participant. Age, race/ethnicity (as reported by the participant), past medical and surgical history, medication use, results of ovarian reserve and hormone laboratory testing, ovarian stimulation parameters, and the information on the number and maturation stage of oocytes were collected. All patients reported in this study had their demographics, medical history, IVF parameters, and outcomes collected and analyzed. However, not all patients had CC and/or FF collected due to logistical reasons (e.g. embryology laboratory volume, staffing, research team availability). This IRB protocol continues to enroll patients to establish a biobank of CCs and FF for future studies (Supplementary Fig. S1).

### Ethical approval

All participants gave written informed consent according to the protocol approved by Northwestern University Institutional Review Board (STU00213161). Of note, it is not standard practice in our clinic to use gonadotropin stimulation in pre-menarchal patients. Ten-year-old peri-pubertal patients underwent ovarian stimulation and oocyte retrieval under a separate IRB protocol (2021-4125).

### Ovarian stimulation and oocyte retrieval protocol

All study participants underwent ovarian stimulation using the GnRH antagonist protocol. Recombinant FSH (Follistim; Organon, Jersey City, NJ, USA, or Gonal-f; EMD-Serono, Rockland, MA, USA) and highly purified HMG (Menopur; Ferring Pharmaceuticals, Saint-Prex, Switzerland) were used for ovarian stimulation and doses were determined based on ovarian reserve at primary physician’s discretion. GnRH antagonist (Ganirelix or Cetrotide; Merck, Rahway, NJ, USA) was added when the lead follicle reached 13 mm or when estradiol levels were above 300 pg/ml per clinic protocol. Ovulation was triggered with HCG (Ovidrel; EMD-Serono or Novarel; Ferring Pharmaceuticals) and/or GnRH agonist trigger (Lupron; AbbVie, North Chicago, IL, USA) at the discretion of the physician, when at least two follicles reached an average size of 20 mm. Transvaginal oocyte retrieval was performed 36 h after the trigger injection.

### Sample collection

Adolescents (n = 19) and oocyte donors (n = 19) had high-quality RNA extracted and sequencing libraries successfully prepared. Of all CC samples available at the time of RNA extraction, only one (adolescent) failed successful RNA extraction and library preparation. For FF cytokines arrays, we analyzed all adolescent FF samples available at the time these arrays were performed (n = 18), however, we only included donors that were 25–30 years old (n = 16) due to the setup of these arrays to maintain a relatively large age gap between adolescent and oocyte donor samples (Supplementary Fig. S1).

**Figure 1.**
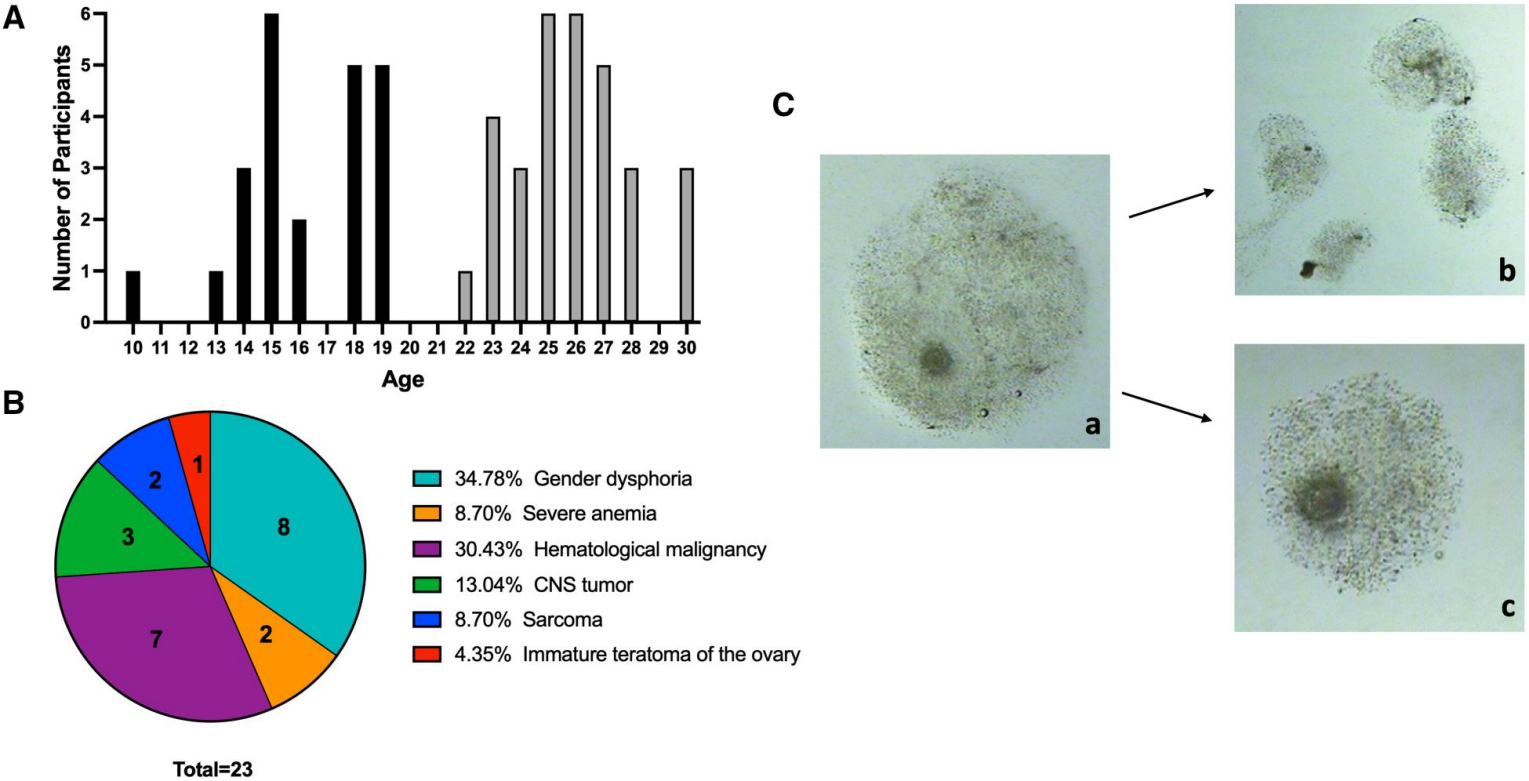
**Participant age and medical diagnosis, and cumulus cell collection.** (**A**) Distribution of age among adolescents (black bars) and oocyte donors (gray bars). (**B**) Medical diagnoses of adolescents. (**C**) Microdissection of cumulus cells from retrieved expanded COCs prior to hyaluronidase treatment: (a) expanded COC before microdissection of cumulus cell masses, (b) trimmed cumulus cell masses (n = 4), (c) COC after microdissection, COC, cumulus–oocyte complex.

CCs were mechanically dissected from cumulus–oocyte complexes (COCs) prior to hyaluronidase treatment to avoid changes in gene expression in the associated cells due to disruption of the extracellular matrix (Fig. 1C) (Spencer *et al.*, 2007; Lelièvre, 2009; Hastings *et al.*, 2019). Our method ensures that the CC transcriptome is preserved in its native state. CC masses from 6–12 COCs per participant were collected. We established that collecting four CC clumps per COC and pooling these clumps from 3 COCs yields optimal RNA (>100 ng total RNA per sample) of good quality (RNA integrity number [RIN] ≥7) for RNA-seq library preparation and sequencing. Following the retrieval, the FF tubes were emptied into a 100-mm petri dish to locate the COCs. Four CC clumps were microdissected from fully expanded COCs (Fig. 1C) measuring around 1500–2000 µm in diameter. We aimed to keep CC clumps consistent in size: ∼250–500 µm. We used a 1-ml syringe with a 25G needle, bent to a 45° angle, which allows for smoother trimming to avoid cutting into the dish while removing the CC clumps. Needles were changed between COCs. The CC clumps were dissected from four quadrants of COCs where possible. Subsequently, they were picked up with a pipette and swiftly rinsed together in a single well of a 4-well IVF plate, each containing 500 µl PBS (Phosphate-buffered saline, Cat No. 20012027, Thermo Fisher Scientific, Waltham, MA, USA) for no more than 2–3 s. Rinsed CC clumps were transferred under the microscope into 0.5-ml microcentrifuge tubes containing 10µl of RNAlater (Cat No. AM7024, Thermo Fisher Scientific) to preserve RNA integrity. Care was taken to avoid expelling excessive PBS into the RNAlater-containing tube to prevent dilution. The microcentrifuge tube containing the four CC clumps was then placed in an icebox and stored at 4°C until collected by the research team. The research team typically picked up the samples within 1- to 2-h post-retrieval, after which the CC tubes were snap frozen at −80°C.

The remaining microdissected COCs were relocated to a corresponding numbered drop dish to monitor oocyte maturity (Fig. 1C). Microdissected COCs were rinsed and held for ∼2 h in Quinn’s Advantage Fertilization Medium supplemented with 5% human serum albumin (Cat Nos ART-1021 and ART-3001, CooperSurgical Fertility Companies, Ballerup, Denmark) in a 37°C, 6% CO_2_, and 5% O_2_ incubator until denudation as per the routine IVF laboratory protocol. After denudation, the maturation stage of the oocyte was determined based on morphology and linked to the collected CC samples. Oocytes arrested at metaphase of meiosis II (MII) were characterized by extrusion of the first polar body (PBI), whereas oocytes that failed to mature and remained arrested at prophase of meiosis I were characterized by an intact nucleus or germinal vesicle (GV oocyte). Oocytes that had undergone germinal vesicle breakdown but lacked PBI (in between prophase I and MII) were referred to as a MI oocyte. Since the maturity of the oocyte influences CC gene expression (Wyse *et al.*, 2020), we ensured to use CCs from only mature (MII) oocytes for further analysis.

FF samples from the two dominant follicles (18–20 mm) of each ovary were collected per participant. Halt protease inhibitor cocktail (Cat No. 87786, Thermo Fisher Scientific) was added at 1 ×  concentration to avoid protein degradation. Cells were pelleted via centrifugation at 380*g* for 10 min at 4°C, and the FF supernatant was transferred to sterile 1.5-ml Eppendorf tubes. Samples were stored at −80°C until further analysis.

### RNA isolation and sequencing

RNA was isolated from CCs combined from three MII oocytes for all study participants in parallel, using an RNAeasy Micro Kit (Cat No. 74004, Qiagen, Valencia, CA, USA). Extracted RNA was quantified with a Qubit fluorometer (Cat No. Q33238, Thermo Fisher Scientific), and RNA quality control was performed with a Agilent 2100 Bioanalyzer RNA 6000 Pico Chip (Cat No. 5067-1513, Agilent Technologies, Santa Clara, CA, USA). Samples with an RIN >7 passed quality control and were used for library preparation (Supplementary Fig. S2) with the Illumina Stranded mRNA Library Prep Kit according to the manufacturer’s instructions (Cat No. 20040532, Illumina Inc., San Diego, CA, USA). This procedure includes mRNA purification and fragmentation, cDNA synthesis, 3′ end adenylation, Illumina adapter ligation, library PCR amplification, and validation. Paired-end sequencing (150 bp) was performed on an S4 flow cell using the lllumina NovaSeq 6000 sequencer (Cat No. 20068232, Illumina Inc) with the production of 20–25 million reads per sample.

### Bioinformatic analysis

The quality of reads, in FASTQ format, was evaluated using FastQC. Reads were trimmed to remove Illumina adapters from the 3′ ends using cutadapt (Spencer *et al.*, 2007). Trimmed reads were aligned to the *Homo sapiens* genome (hg38) using STAR (Dobin *et al.*, 2013). Read counts for each gene were calculated using htseq-count (Anders *et al.*, 2015), and in conjunction with a gene annotation file consisted of 58 396 genes for hg38 obtained from Ensembl (Martin *et al.*, 2023). Normalization and differential expressions were calculated using DESeq2 which employs the Wald test (Love *et al.*, 2014). The cutoff for determining significantly differentially expressed genes (DEGs) was an FDR-adjusted *P*-value <0.05 using the Benjamini–Hochberg method. Enrichment analysis was performed using downregulated and upregulated DEGs via the Metascape online platform (Zhou *et al.*, 2019) to identify differences in gene ontology (GO) biological pathways across two groups. The top 20 significantly enriched GO terms were plotted using RStudio version 4.3.1 (R Foundation for Statistical Computing, Vienna, Austria; https://www.R-project.org/).

### Cytokine antibody array

FF samples from adolescent and oocyte donors were thawed and a 2-fold dilution was performed using the blocking buffer provided in the Human Cytokine Array C5 as previously described (Ray Biotech Inc, Norcross, GA, USA) (Machlin *et al.*, 2021). Diluted samples (1 ml) were run as duplicates in parallel on arrays according to the manufacturer’s instructions. The arrays were visualized by chemiluminescence using a BioRad ChemiDoc Imaging System (Cat No. 12003153, Bio-Rad Laboratories Inc., Hercules, CA, USA). The resulting chemiluminescence data were quantified using the Protein Array Analyzer plugin for FIJI software (Schindelin *et al.*, 2012). The relative intensity units (RU) of 80 cytokines were averaged between duplicate FF samples with the background subtracted. The RU of these 80 cytokines was compared between adolescents and oocyte donors.

### Statistical analysis

The normal distribution of the data was evaluated with the Shapiro–Wilk and Kolmogorov–Smirnov tests. Analysis between two groups of continuous variables was performed with unpaired two-sided Student’s *t*-test or Mann–Whitney *U*-test depending on distribution. Categorical variables were analyzed with Fisher’s exact test or chi-square test. Analysis between the three groups of continuous variables was performed with ordinary one-way ANOVA or Kruskal–Wallis test depending on the distribution. Tukey’s or Dunn’s multiple comparisons test was used for *post hoc* analysis. Data are presented as mean ± SEM. *P*-values <0.05 were considered statistically significant. GraphPad Prism version 9.0.1 (Boston, MA, USA, www.graphpad.com) was used for statistical analysis.

## Results

### Demographic and clinical characteristics of participants

Adolescent participants were on average a decade younger than oocyte donors (16.6 ± 0.5, 10–19 years old, n = 23 vs 26.3 ± 0.4, 22–30 years old, n = 31, *P* < 0.0001) (Table 1, Fig. 1A). Thirteen adolescents were diagnosed with cancer prior to fertility preservation, whereas 10 adolescents had a non-cancer diagnosis, including gender dysphoria (n = 8) and severe anemia requiring stem cell therapy (n = 2) (Fig. 1B). Five of 13 patients had received previous chemo- and/or radiotherapy. BMI (25.2 ± 1.35 vs 24.2 ± 0.45 kg/m^2^, *P* = 0.83) and race/ethnicity were similar across two groups of patients. Only one adolescent patient (10 years old) was pre-menarchal, whereas the rest (n = 22) were at least 2 years post-menarchal. Anti-Müllerian hormone (AMH) levels and antral follicle count (AFC) were significantly higher in oocyte donors compared to adolescents (AMH: 6.6 ± 0.66 ng/ml vs 3.3 ± 0.47 ng/ml and AFC: 26.3 ± 1.8 vs 16.8 ± 1.2, *P* < 0.0001) (Table 1). In line with this, oocyte donors required lower total gonadotropin doses (4131 ± 274 vs 5322 ± 427, *P* = 0.024) and had higher peak estradiol (E_2_) levels (3820 ± 256 vs 2450 ± 256, *P* = 0.0006) compared to adolescents. Luteal phase start (34.78% vs 3.23%, *P* = 0.003) and abdominal ultrasound monitoring (65% vs 0%, *P* < 0.0001) were more common for adolescents. Duration of stimulation (11.3 ± 0.3 vs 11.3 ± 0.2, *P* = 0.92), the number of monitoring visits (6.1 ± 0.2 vs 6.6 ± 0.2, *P* = 0.14), and the number of retrieved oocytes (MII: 20.2 ± 2.6 vs 24.4 ± 2.3, *P* = 0.15; MI: 1.3 ± 0.3 vs 2.2 ± 0.4, *P* = 0.09 and GV: 4.5 ± 1.1 vs 2.8 ± 0.6, *P* = 0.26) were similar between groups. The number of degenerate gametes (1.6 ± 0.4 vs 0.5 ± 0.2, *P* < 0.05) as well as empty zonae (EZ) collected (2.0 ± 0.6 vs 0.4 ± 0.2, *P* < 0.01) at retrieval were higher for adolescent participants.

**Table 1.** Demographics and IVF cycle characteristics.

	Adolescents	Donors	*P*-value
	(n=23)	(n=31)	
Age (years)	16.6±0.5	26.3±0.4	<0.0001**
BMI (kg/m^2^)	25.2±1.35	24.2±0.45	0.8317
Race/ethnicity (number of participants)			0.3717
Caucasian	12	25	
African American	3	2	
Asian	1	2	
Middle Eastern	1	0	
Hispanic	1	1	
Multi-racial	3	1	
AMH (ng/ml)	3.3±0.47	6.6±0.66	<0.0001**
Antral follicle count	16.8±1.2	26.3±1.8	<0.0001**
Luteal phase start	34.78%	3.23%	0.003**
Duration of stimulation (days)	11.3±0.3	11.3±0.2	0.9248
Number of monitoring visits (days)	6.1±0.2	6.6±0.2	0.1382
Type of ultrasound			<0.0001**
Transvaginal (%)	34.78%	100.00%	
Transabdominal (%)	65.22%		
Total gonadotropin dose (IU)	5322±427	4131±274	0.024*
Peak estradiol (pg/ml)	2450±256	3820±256	0.0006**
Number of oocytes	29.9±3.8	32.7±2.3	0.5088
Number of MII oocytes	20.2±2.6	24.4±2.3	0.1461
Number of MI oocytes	1.3±0.3	2.2±0.4	0.0896
Number of GVs	4.5±1.1	2.8±0.6	0.2606
Number of degenerated oocytes at retrieval	1.6±0.4	0.5±0.2	0.0195*
Number of EZs	2.0±0.6	0.4±0.2	0.0007**

Values are presented as mean±SEM. Values in bold text are significant.

* *P* < 0.05.

** *P* < 0.01.

BMI, body mass index; AMH, anti-Müllerian hormone; MII oocytes, mature metaphase II arrested oocytes; MI oocytes, immature oocytes between GV and MII stage; GVs, immature oocytes with germinal vesicles; EZs, zona pellucida devoid of an oocyte.

### The CC transcriptome of adolescents reveals differentially regulated biological pathways compared to oocyte donors

**Figure 2.**
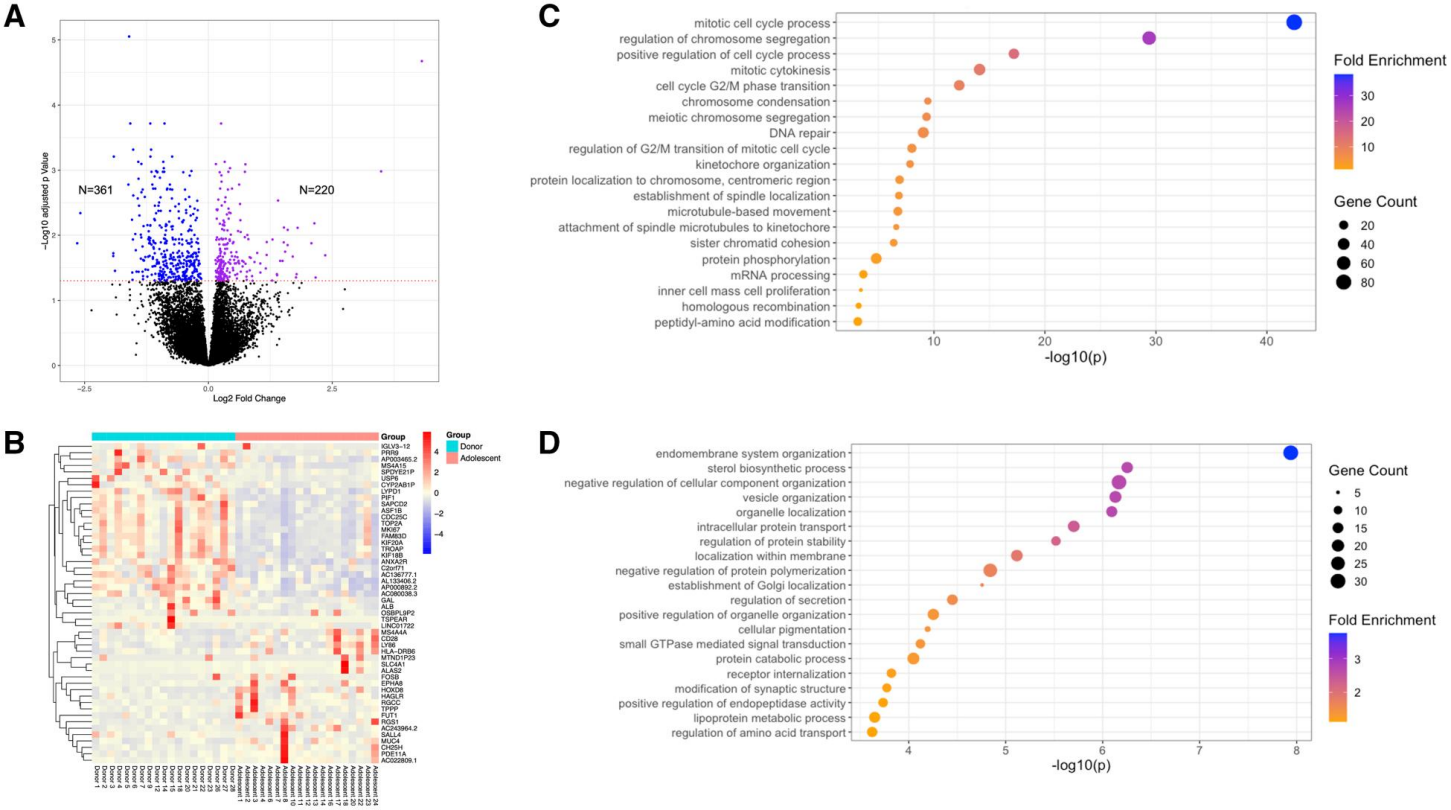
**Comparative RNA-seq analysis of cumulus cells collected from adolescents and oocyte donors.** (**A**) Volcano plot with downregulated (n = 361) and upregulated DEGs (n = 220) in adolescents (n = 19) compared to donors (n = 19) (dashed red line—adjusted *P* < 0.05). (**B**) Heatmap with the top 50 DEGs with the highest fold change between two age groups. (**C**) Top 20 significantly downregulated GO terms (biological pathways) in adolescents compared to donors. (**D**) Top 20 significantly upregulated GO terms (biological pathways) in adolescents compared to donors. DEGs, differentially expressed genes; GO, gene ontology.

Participants included in the CC transcriptome analysis had on average an 8 year age gap between the groups (16.4 ± 0.5 years old, n = 19 vs 24.1 ± 0.6 years old, n = 19) (Supplementary Fig. S3). Unsupervised hierarchical clustering and principal component analysis (PCA) did not demonstrate clear clustering of CC transcriptomes from adolescents and oocyte donors indicating overall similar CC biology in these age groups, which is not surprising given the highly differentiated nature of these cells (Supplementary Fig. S4). However, comparative transcriptomic analysis revealed 581 DEGs (Fig. 2A), of which 361 genes were downregulated and 220 were upregulated in CCs of adolescents compared to oocyte donors (Fig. 2A and B). Combined GO analysis, considering all DEGs at once, revealed significant differences in genes enriched in biological pathways related to cell cycle and cell division processes between adolescents and oocyte donors. Mitotic cell cycle process (GO: 1903047, *P* = 3.8 × 10^−38^), regulation of cell cycle process (GO: 0010564, *P* = 3.8 × 10^−26^), and positive regulation of cell cycle process (GO: 0090068, *P* = 9.7 × 10^−19^) emerged as the top three significantly different biological pathways (Supplementary Fig. S5). When down- and upregulated genes were considered separately, genes enriched in biological pathways involved in cell cycle and cell division processes were significantly downregulated in adolescents compared to oocyte donors. Mitotic cell cycle process (GO: 0051983, *P* = 4.1 × 10^−30^), regulation of chromosome segregation (GO: 0051983, *P* = 3.5 × 10^−43^), positive regulation of cell cycle process (GO: 0090068, *P* = 4 × 10^−30^), mitotic cytokinesis (GO: 0000281, *P* = 7.7 × 10^−15^), cell cycle G2/M phase transition (GO: 0044839, *P* = 5.2 × 10^−13^), and chromosome condensation (GO: 0030261, *P* = 3.6 × 10^−10^) were the top downregulated biological pathways (Fig. 2C). Transcripts in pathways involved in cellular communication, intracellular transport processes, and organelle organization were more abundant in CCs of adolescents compared to oocyte donors. Endomembrane system organization (GO: 0010256, *P* = 1.2 × 10^−8^), sterol biosynthetic process (GO: 0016126, *P* = 5.6 × 10^−7^), negative regulation of cellular component organization (GO: 0051129, *P* = 6.8 × 10^−7^), vesicle organization (GO: 0016050, *P* = 7.4 × 10^−7^), organelle localization (GO: 0006886, *P* = 8 × 10^−7^), and intracellular protein transport (GO: 0016050, *P* = 1.9 × 10^−6^) were the top upregulated biological pathways (Fig. 2D).

Several subgroup analyses were performed to ascertain the impact of age and cancer diagnosis on DEGs and altered biological pathways. Although the number of DEGs between CCs from adolescents and oocyte donors increased to 1069 when the analysis was limited to individuals <16 years old (median age of participants), the biological pathways affected overall remained similar to the original analysis (Supplementary Fig. S6). Furthermore, CC transcriptomic analysis of adolescents ≥16 years old (n = 10) vs <16 (n = 9) revealed only 41 DEGs (Supplementary Fig. S7). These results demonstrate that biological pathways appear dysregulated in CCs of adolescents of all ages compared to oocyte donors in our study. Importantly, no DEGs were identified in CCs of adolescents with cancer (n = 11) compared to adolescents without cancer (n = 8) (Supplementary Fig. S8). Removing one possible outlier (non-cancer, far right on PCA plot in Supplementary Fig. S8) from this analysis also did not lead to clustering of cancer and non-cancer patients, and DEG analysis showed 11 genes with differential expression (Supplementary Fig. S9), indicating that the observed molecular differences are most likely attributable to age and not the underlying cancer diagnosis. Unsupervised hierarchical clustering and PCA also did not reveal distinct clustering patterns of CC transcriptomes across the three groups: adolescents with cancer, adolescents without cancer, and oocyte donors (Supplementary Fig. S10; 10yo patient excluded).

### Follicular fluid of adolescents is more pro-inflammatory compared to oocyte donors

**Figure 3.**
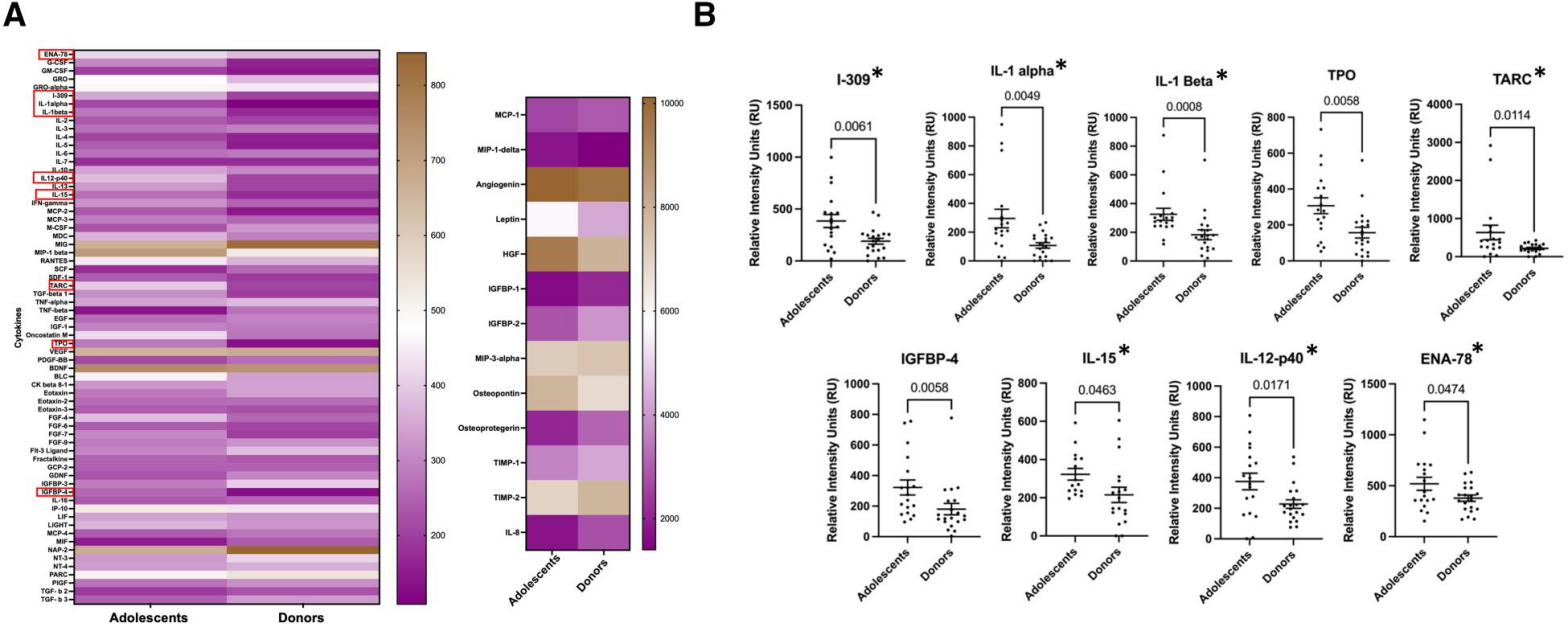
**Follicular fluid levels of cytokines in adolescents and oocyte donors.** (**A**) Heatmap of follicular fluid levels of 80 cytokines in adolescents and oocyte donors with nine of them significantly different between the two groups (red boxes). (**B**) Seven of these nine significantly different cytokines have pro-inflammatory roles (asterisks).

FF cytokine analysis of adolescents (16.7 ± 0.6, 10–19 years old, n = 18) and oocyte donors (27.3 ± 0.4, 25–30 years old, n = 16) (Supplementary Fig. S11) revealed altered levels of nine cytokines (Fig. 3A). All nine cytokines demonstrated higher levels in FF of adolescents compared to oocyte donors: interleukin-1 alpha (IL-1α; 2-fold), interleukin-1 beta (IL-1β; 1.7-fold), I-309 (2-fold), interleukin-15 (IL-15; 1.6-fold), thymus and activation-regulated chemokine (TARC; 1.9-fold), thrombopoietin (TPO; 2.1-fold), insulin-like growth factor (IGF) binding protein-4 (IGFBP-4; 2-fold), the p40 Subunit of interleukin-12 (IL-12-p40; 1.7-fold), and epithelial neutrophil-activating protein 78 (ENA-78; 1.4-fold) (Fig. 3B). Interestingly, seven of these nine cytokines have pro-inflammatory roles (Miller and Krangel, 1992; Trinchieri, 1995; Gilchrest *et al.*, 2003; Persson *et al.*, 2003; Perera *et al.*, 2012; Santana Carrero *et al.*, 2019; Galozzi *et al.*, 2021; Lupancu *et al.*, 2023), suggesting a more pro-inflammatory environment in the FF of the adolescents compared to oocyte donors. The other two, IGFBP-4 and TPO, were shown to be important in the regulation of IGF action (Laursen *et al.*, 2007) and in the production of platelets (Hitchcock *et al.*, 2021), respectively. Enrichment analysis was performed for the nine cytokines that were significantly different between adolescent and oocyte donors. Cytokine-mediated signaling pathway (GO: 0019221, *P* = 1.7 × 10^−12^), cellular response to cytokine stimulus (GO: 0071345, *P* = 1.7 × 10^−10^), neutrophil chemotaxis (GO: 0030593, *P* = 5.9 × 10^−9^), neutrophil migration (GO: 1990266, *P* = 1.0 × 10^−8^), and positive regulation of T cell proliferation (GO: 0042102, *P* = 1.6 × 10^−8^) were the top five significantly upregulated biological pathways in adolescents compared to oocyte donors (Supplementary Fig. S12).

None of these nine cytokines demonstrated altered levels in FF of adolescents with cancer (n = 12) compared to those without a cancer diagnosis (n = 6) (Supplementary Fig. S13A). FF of adolescent patients with cancer had decreased levels of nine cytokines (G-CSF, EGF, FGF-7, BDNF, BLC, LIGHT, MCP-2, IL-7, TGF-beta 1) and increased levels of one cytokine (MCP-1) compared to FF of adolescents without cancer (Supplementary Fig. S13B). This demonstrates that increased levels of pro-inflammatory cytokines in FF of adolescents are likely due to younger age and not the cancer diagnosis.

In addition, we conducted an analysis among three groups: adolescents with and without cancer and oocyte donors, which revealed significantly different FF levels of 11 cytokines (Supplementary Fig. S14A). Of the 11 cytokines, nine were significantly higher in adolescents compared to oocyte donors. The remaining two were only different between adolescents with and without cancer, (LIGHT; higher in non-cancer and MCP-1; higher in cancer) (Supplementary Fig. S14B). Three pro-inflammatory cytokines (IL-1β, IL-1α, TARC for adolescents with cancer and IL-1β, I-309, oncostatin-M for adolescents without cancer) demonstrated significantly higher levels in FF of adolescents compared to oocyte donors. IFGBP4, TPO, BDNF, and FGF-7 demonstrated higher FF levels in adolescents without cancer compared to donors. BDNF and FGF-7 levels were also significantly higher in FF of adolescents without cancer compared to the adolescents with cancer.

## Discussion

Oocyte cryopreservation is an essential fertility preservation option for adolescents and young adults. However, the quality and reproductive potential of the gametes in this age group are understudied despite concerns about potential suboptimal gamete quality (Franasiak *et al.*, 2014; Gruhn *et al.*, 2019). Moreover, the outcomes of pregnancies with these oocytes, as well as the health of future generations, are unknown. Our unbiased molecular analysis of the immediate oocyte microenvironment, in the largest cohort of adolescents to date, revealed differentially regulated biological pathways in CCs and a more pro-inflammatory signature in FF compared to oocyte donors.

Egg quality is suboptimal during mammalian juvenescence (Mirskaia and Crew, 1931; Koenig and Stormshak, 1993; Wallen and Zehr, 2004; Lechniak *et al.*, 2007; Duncan, 2017; Kusuhara *et al.*, 2021), and this phenomenon appears to be conserved in humans. Human oocytes obtained from very young individuals in both IVF cycles and ovarian tissue cryopreservation cases display higher rates of aneuploidy (Franasiak *et al.*, 2014; Gruhn *et al.*, 2019). An altered oocyte microenvironment with increased pro-inflammatory cytokine levels in FF and differential gene expression in CCs may be reflective of the higher aneuploidy observed in a very young population. On the other hand, this altered microenvironment may negatively impact oocyte competence. This represents the classic causality dilemma and future studies should untangle cause and effect relationships. Given the importance of CCs in oocyte cytoplasmic maturation (Mori *et al.*, 2000; Eppig *et al.*, 2005; Gilchrist *et al.*, 2008; Su *et al.*, 2009; Valsangkar and Downs, 2013; Ikeda and Yamada, 2014; Dumesic *et al.*, 2015; Dell’Aversana *et al.*, 2021), we speculate that, in addition to the increased aneuploidy, oocyte cytoplasmic maturation is also suboptimal in adolescents.

CCs support the growth and metabolism of the associated oocyte (Gilchrist *et al.*, 2004; Xie *et al.*, 2023), provide valuable insights into the quality of the associated gamete, and can be readily sampled in IVF cycles. In preovulatory follicles, LH surge triggers synthesis and secretion of epidermal growth factor (EGF)-like mediators from mural granulosa cells. As oocyte and CCs lack LH receptors, these factors, including amphiregulin, epiregulin, and beta-cellulin, propagate the LH stimulus down to COCs (Park *et al.*, 2004). These mediators augment CC proliferation in mice, goat, and pigs (Cakmak *et al.*, 2016; Baszary and Moniharapon, 2020; Zhang *et al.*, 2022). A study in a bovine model demonstrated that the interruption of the communication axis between oocyte and CCs by dissociation of CCs from COCs results in decreased CC proliferation and expansion, ultimately triggering apoptosis in both cell types (Luciano *et al.*, 2000). Downregulated expression of proliferation pathways in adolescent CCs may indicate an abnormal response to, or disordered signaling of, EGF-like growth factors, and the upregulation of pathways related to cellular organization, transport, and intercellular communication may signify a compensatory mechanism. In fact, some of the genes involved in EGF signaling demonstrate altered transcript levels in CCs of adolescents compared to oocyte donors (i.e. *TMEFF1, ZGPAT, MAPK8IP3, PBK*) which warrants further investigation. Whether these molecular differences lead to impaired cumulus expansion, similar to that observed with advanced reproductive age (Babayev *et al.*, 2023), also remains to be investigated.

It is important to note that the CCs and FF utilized in this study were obtained from ovarian stimulation cycles. Therefore, the CCs were luteinized. Biological differences are expected between these and naïve samples. To understand the fundamental biological differences between the immediate oocyte microenvironment across age groups, naïve cells represent the ideal samples to avoid the confounding effects of ovarian stimulation and luteinization on results. However, in the context of fertility preservation, to understand how adolescent oocytes obtained in ovarian stimulation cycles differ from adults, CCs and FF from stimulated cycles provide clinically relevant samples, as these are the microenvironment of the oocytes that are planned to be used for future reproduction. Although their characteristics are expected to be different from the naïve samples, they provide insights into the quality of the cryopreserved oocytes from controlled ovarian stimulation cycles.

The differences in the phase of the menstrual cycle at the start of hormonal stimulation, gonadotropin doses, and peak estradiol levels (as donors required less gonadotropin stimulation and had higher estradiol levels at the time of trigger) may have also contributed to the observed molecular disparities between our groups. Additionally, although no donors were previously diagnosed with PCOS, many used birth control pills or progestin intrauterine devices for contraception. Therefore, we cannot exclude PCOS diagnosis in this group which may also confound our results. However, other demographic (e.g. BMI, race/ethnicity) and oocyte retrieval parameters (e.g. trigger criteria, number of oocytes at different stages of maturation) were similar between the groups.

We did not control for the follicle size due to logistical reasons. All COCs were retrieved from large, >16mm follicles. CC transcriptomes of individual COCs may vary. Although collecting COCs from similar size follicles in both groups, ensuring that the CCs only associated with mature oocytes were used for downstream analysis, and combining cumulus clumps from three COCs per patient mitigates the impact of inter-COC transcriptome differences of the same individual on our results, we cannot exclude that this had at least some effect. Similarly, FF samples were collected from large antral follicles (18–20 mm) in both groups. We also cannot exclude that small (2 mm or less) differences in antral follicle size may affect the FF cytokine profile. However, given that similar size follicles were retrieved in adolescent and donors groups using the same methodology, inter-individual differences rather than inter-follicular variations are more likely to be responsible for the observed differences.

Another limitation of our study is the reliance on the analysis of CC transcriptome, and future studies focusing on targeted proteomic analysis of these differentially regulated pathways may elucidate whether observed alterations persist at the protein level. However, proteomic studies also have challenges and limitations, including sufficient sample availability and issues with the contamination of proteins from the FF and media used in oocyte retrieval procedures.

Importantly, no DEGs were noted between adolescents with cancer compared to those without. This suggests that the observed molecular differences are likely attributed to age rather than the presence of cancer. We also cannot exclude that the previous chemo- and/or radiotherapy (5/13 adolescent patients) may have affected our results. However, given there were no DEGs in CCs of adolescents with cancer vs those without cancer, this is unlikely to be a major contributor to the dysregulated gene expression observed in the adolescent group compared to the oocyte donors. Interestingly, the affected biological pathways remained generally similar when the analysis was limited to adolescents <16 years old compared to oocyte donors, and very few DEGs were observed between adolescents ≥16 years old compared to those <16 years old. This suggests that oocyte cryopreservation in an adolescent population may have similar prognosis across an age spectrum of 10 to 19 years old. However, larger studies directly examining the quality of oocytes are needed for definitive conclusions.

FF reflects the metabolism of the surrounding granulosa and CCs and plays a crucial role in supporting the maturation and development of the oocyte within the antral follicle (Edwards, 1974). Reproductive aging is associated with inflammation, oxidative damage, ovarian fibrosis, and increased stiffness in the ovarian stroma (Amargant *et al.*, 2020; Wang *et al.*, 2021; Umehara *et al.*, 2022; Babayev *et al.*, 2023). Similarly, FF demonstrates a more fibro-inflammatory cytokine signature (IL-3, IL-7, IL-15, TGFβ1, TGFβ3, and MIP-1) with aging (Machlin *et al.*, 2021). Our study demonstrated that FF of adolescents has higher levels of pro-inflammatory cytokines (IL-1α, IL-1β, I-309, IL-15, TARC, IL-12-p40, ENA-78) compared to oocyte donors. The majority of these cytokines were unique to the adolescent population and notably, only one of these cytokines, IL-15, was shared with women of advanced reproductive age, as reported in the previous study (Machlin *et al.*, 2021). Although these data may indicate the tendency that a more pro-inflammatory milieu exists at both ends of the age spectrum, the levels of individual cytokines and mechanisms may differ. Cancer is associated with inflammation (Singh *et al.*, 2019). However, none of the afore mentioned cytokines had significantly different levels in FF of adolescents with cancer compared to those without a cancer diagnosis. This indicates that the increase in pro-inflammatory cytokine levels in FF of adolescents compared to donors is likely attributed to age rather than cancer diagnosis.

The success of the bidirectional regulatory loop between oocytes and CCs, and the oocyte’s intrinsic ability to govern its own microenvironment by oocyte-secreted factors are crucial characteristics contributing to oocyte quality (Corn *et al.*, 2005). Therefore, more pro-inflammatory FF environment and dysregulation in CC proliferation and organelle organization pathways could possibly be attributed to the suboptimal oocyte quality in adolescents. Notably, increasing aneuploidy rates with decreasing age in children and young adults, is a consistent observation regardless of the oocyte source. Gruhn *et al.*, 2019 demonstrated that aneuploidy rates increase with decreasing age, below the age of 27 years, in oocytes obtained from ovarian tissue cryopreservation samples, similar to those obtained with ovarian stimulation cycles (Gruhn *et al.*, 2019). Ovarian tissue cryopreservation is still the preferred fertility preservation option for prepubertal adolescents, while ovarian stimulation is preferred for post-pubertal patients. Our study raises further concerns regarding relatively poorer oocyte quality at the young end of the age spectrum, which potentially extends beyond aneuploidy and affects cytoplasmic maturation given the alterations in the CC gene expression. These findings have implications for counseling of adolescent patients undergoing fertility preservation about their reproductive potential as it relates to the number of required cryopreserved oocytes. The optimal number of oocytes that need to be cryopreserved for a reasonable chance of live birth is likely higher for adolescents relative to older individuals. Clinicians should take this into account when counseling their patients.

## Supplementary Material

hoae047_Supplementary_Data

## Data Availability

The original high-throughput sequencing data underlying this article are available in Gene Expression Omnibus (GEO) database and can be accessed with the accession number GSE265995.
