# Supplementary material for: The oocyte microenvironment is altered in adolescents compared to oocyte donors
Source: Hum Reprod Open. 2024 Aug 6;2024(3):hoae047. doi: 10.1093/hropen/hoae047 (PMC11361810; doi:10.1093/hropen/hoae047)
Supplement: hoae047_Supplementary_Data [file hoae047_supplementary_data.pdf]

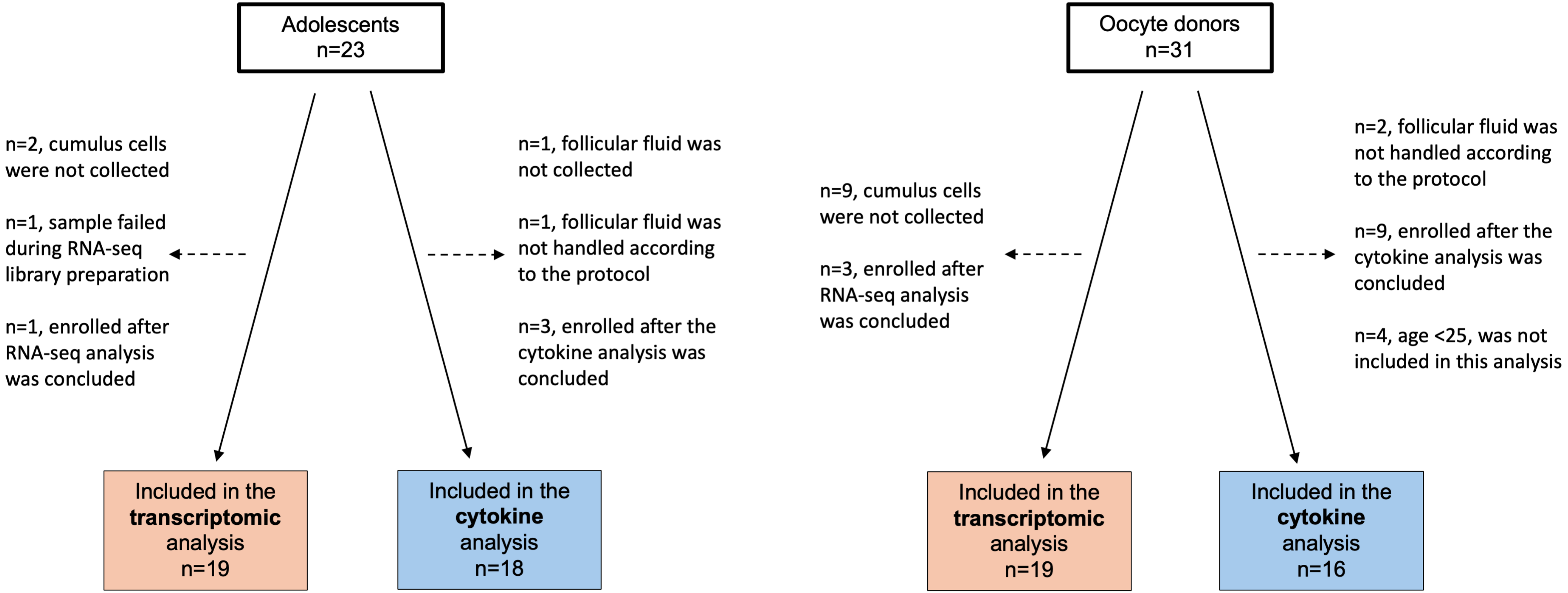

Supplementary Figure S1. Study population and sample collection scheme.

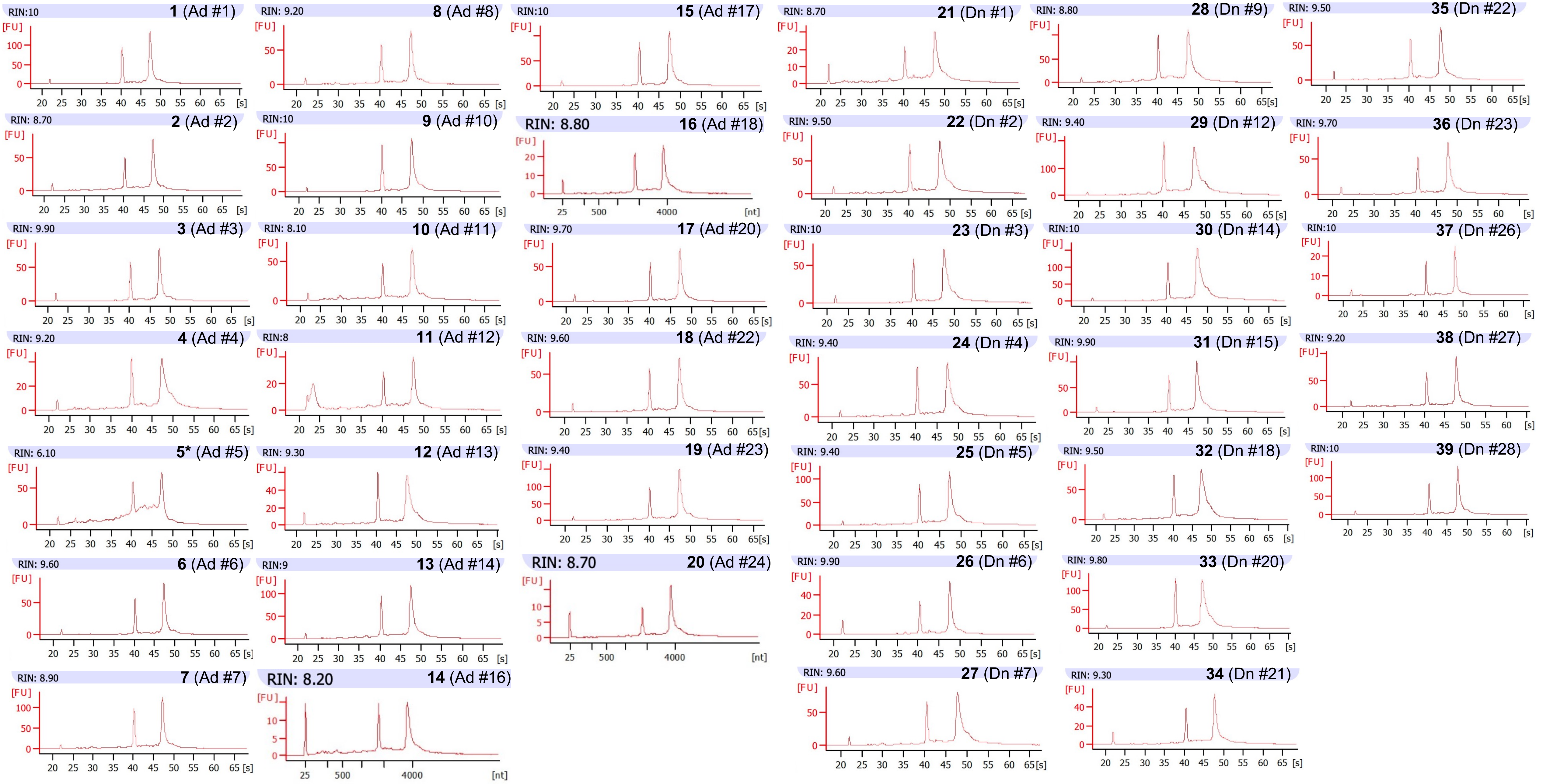

Supplementary Figure S2. Adolescents' (Ad; n=20) and oocyte donors' (Dn; n=19) cumulus cells RNA quality assessment by the Agilent 2100 Bioanalyzer. *Only one sample (Ad #5) had RNA Integrity Number (RIN) <7 and was excluded from library preparation and sequencing.

A

	Adolescents (n=19)	Donors (n=19)	P value
**Age** (years)	16.4 ± 0.5	24.1 ± 0.6	**<0.0001****
**BMI** (kg/m^2)	25.8 ± 1.54	24.1 ± 0.56	0.598
**Race/ethnicity** (number of participants)			0.4662
Caucasian	9	15	
African American	3	2	
Asian	1	1	
Middle Eastern	1	0	
Hispanic	1	1	
Multi-racial	2	0	
**AMH** (ng/mL)	3.4 ± 0.56	6.9 ± 0.89	**0.0009****
**Antral follicle count (AFC)**	16.1 ± 1.2	27.8 ± 2.8	**<0.0001****
**Luteal start**	42.11%	5.26%	**0.0188***
**Duration of stimulation** (days)	11.3 ± 0.3	11.5 ± 0.2	0.3663
**Number of monitoring visits** (days)	6.2 ± 0.2	6.8 ± 0.3	0.099
**Type of USG**			**<0.0001****
Transvaginal%	31.58%	100.00%	
Transabdominal%	68.42%		
**Total gonadotropin dose** (IU)	5341 ± 442	3968 ± 303	**0.0171***
**Peak estradiol** (pg/mL)	2422 ± 303	4034 ± 356	**0.0009****
**Number of oocytes**	31 ± 4.1	31.8 ± 2.7	0.8733
**Number of MIIs**	21 ± 2.8	25.8 ± 2.6	0.124
**Number of MIs**	1.5 ± 0.3	2.6 ± 0.4	**0.0274***
**Number of GVs**	4.1 ± 1.2	2.8 ± 0.6	0.7095
**Number of degenerated oocytes at retrieval**	1.7 ± 0.5	0.2 ± 0.1	**0.0058****
**Number of EZs**	2.3 ± 0.7	0.4 ± 0.2	**0.0037****

Values are presented as mean ± SEM. *P<0.05 and **P<0.01 is significant. BMI = body mass index; AMH = Anti-Mullerian hormone; MIIs = mature metaphase II arrested oocytes; MIs = immature oocytes between GV and MII stage. GVs = immature oocytes with germinal vesicle; EZs = zona pellucida devoid of an oocyte.

B

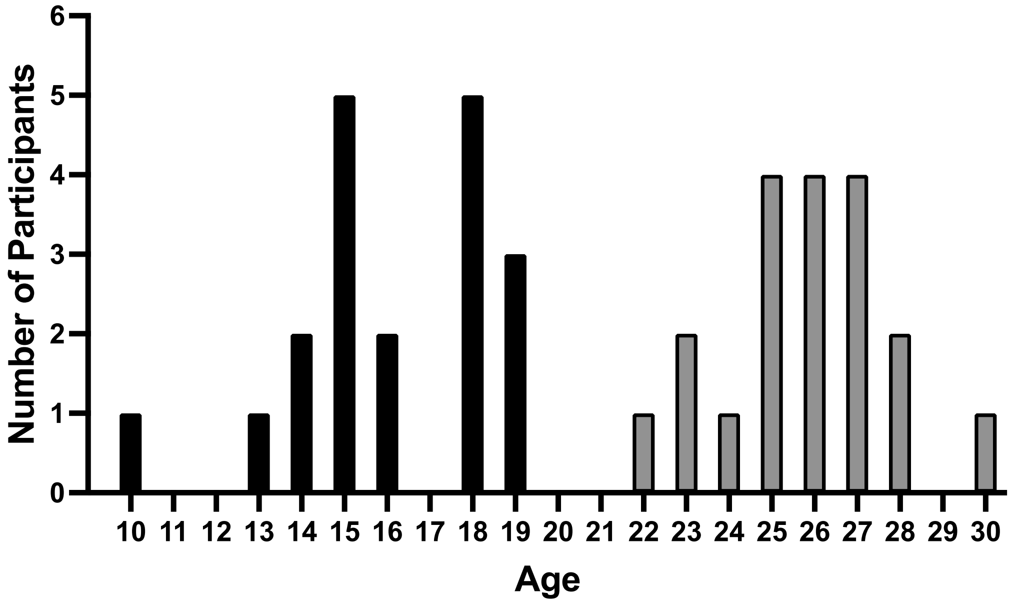

C

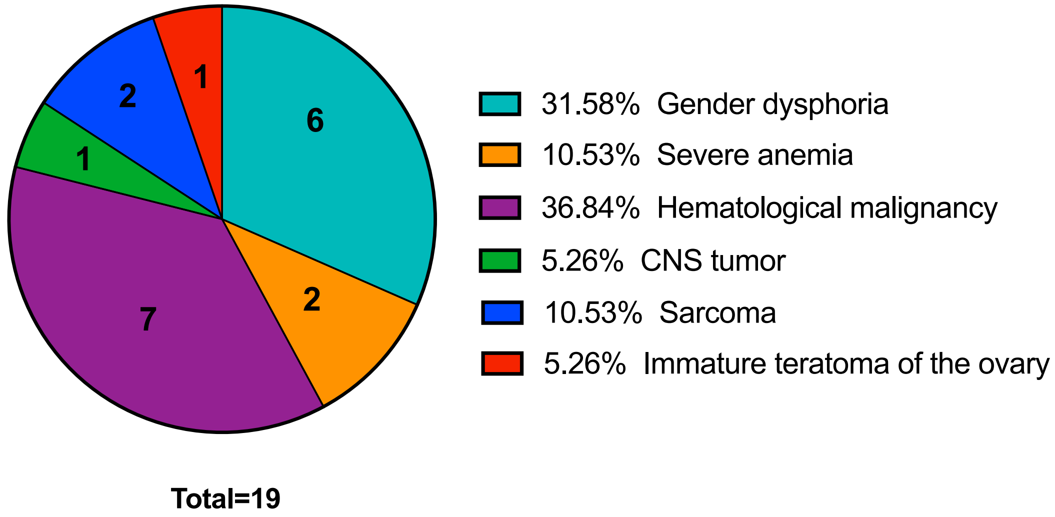

Supplementary Figure S3. Demographics and IVF cycle characteristics (A), age: adolescents (black bars), oocyte donors (grey bars) (B) of participants, and medical diagnoses of adolescents (C) included in cumulus cell transcriptomic analysis.

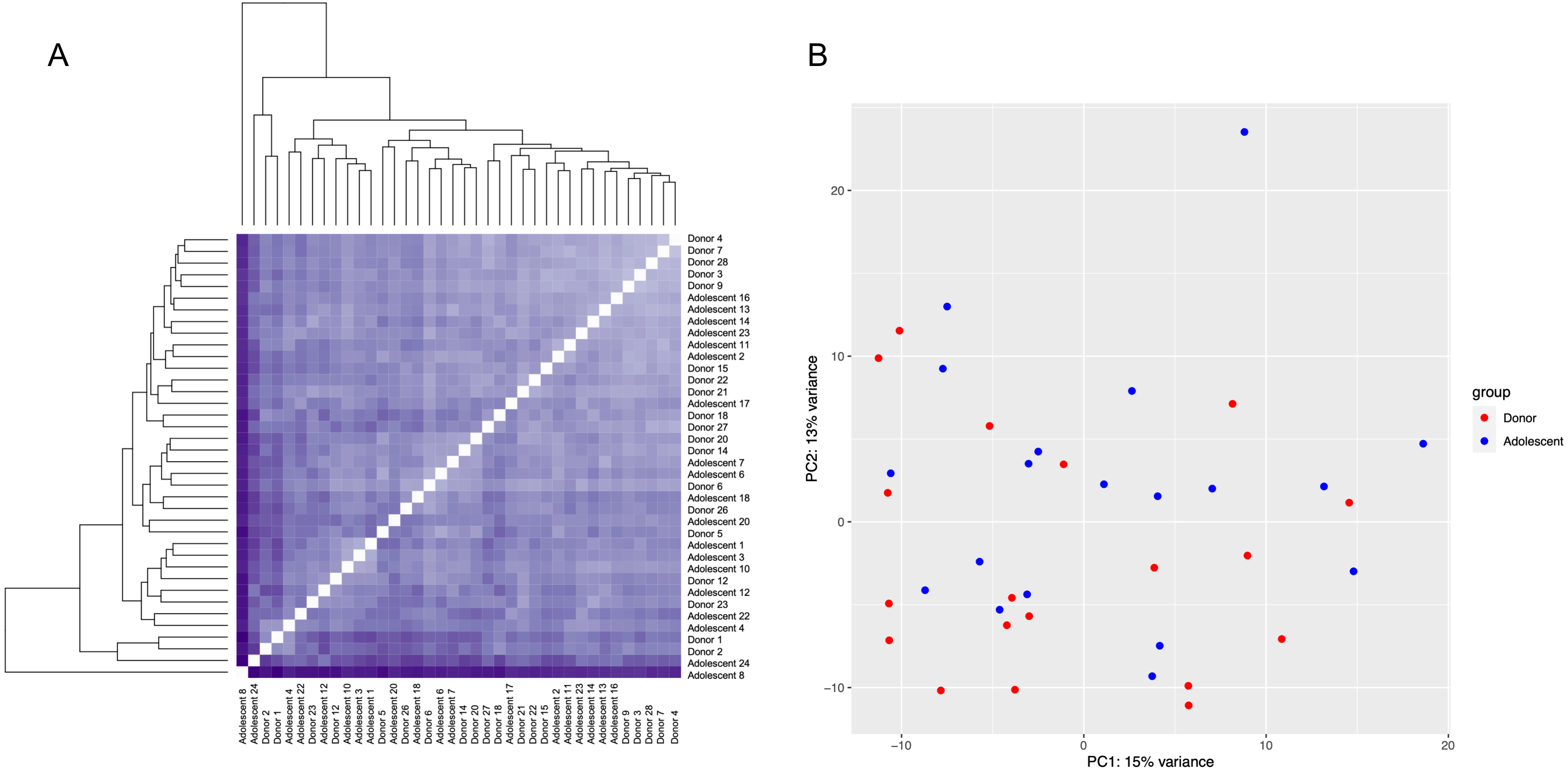

Supplementary Figure S4. RNA-seq analysis of cumulus cells collected from adolescents and oocyte donors.
A) Unsupervised hierarchical clustering B) Principal component analysis

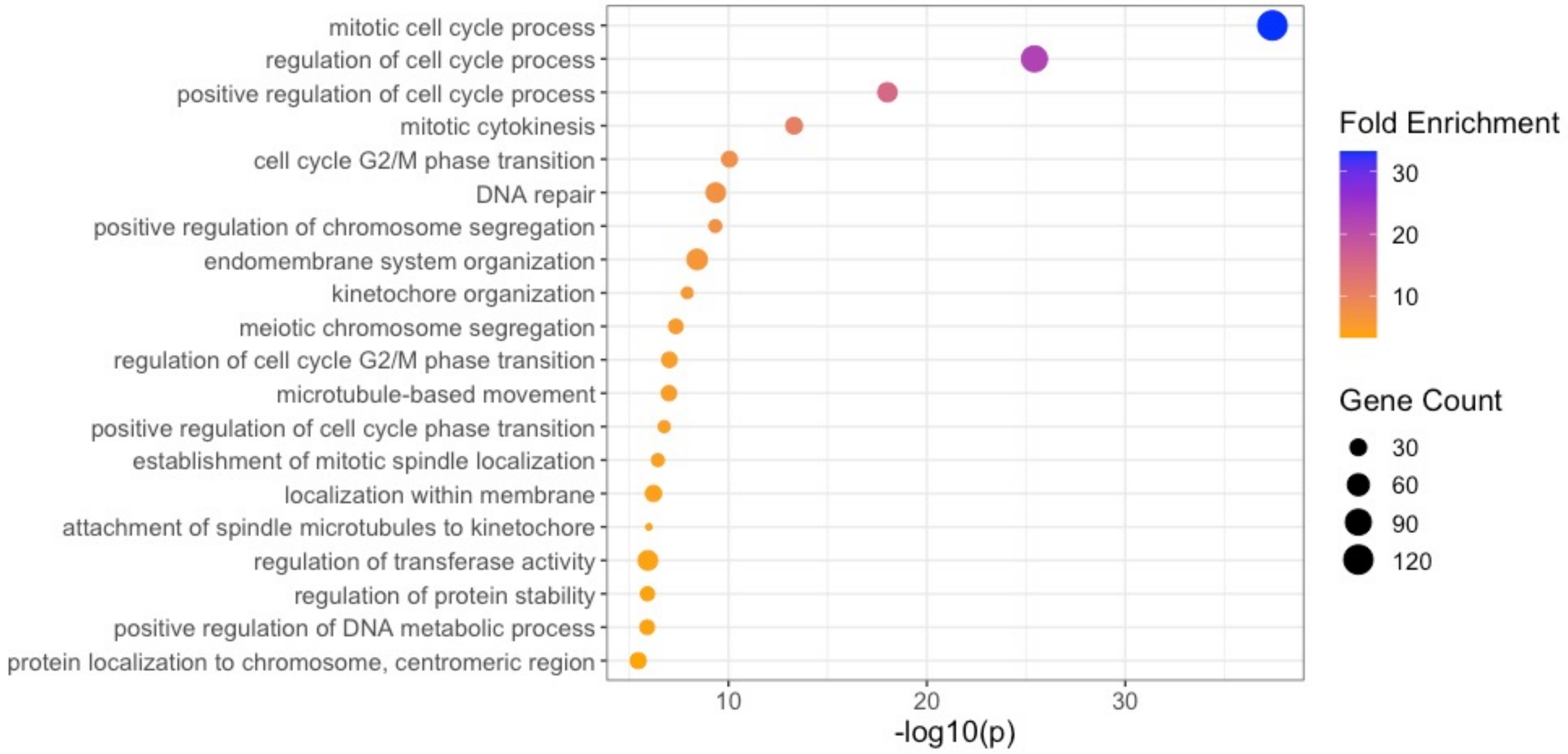

Supplementary Figure S5. Top 20 significantly different GO terms (biological pathways) based on RNA-seq analysis of cumulus cells collected from adolescents compared to oocyte donors. GO: gene ontology.

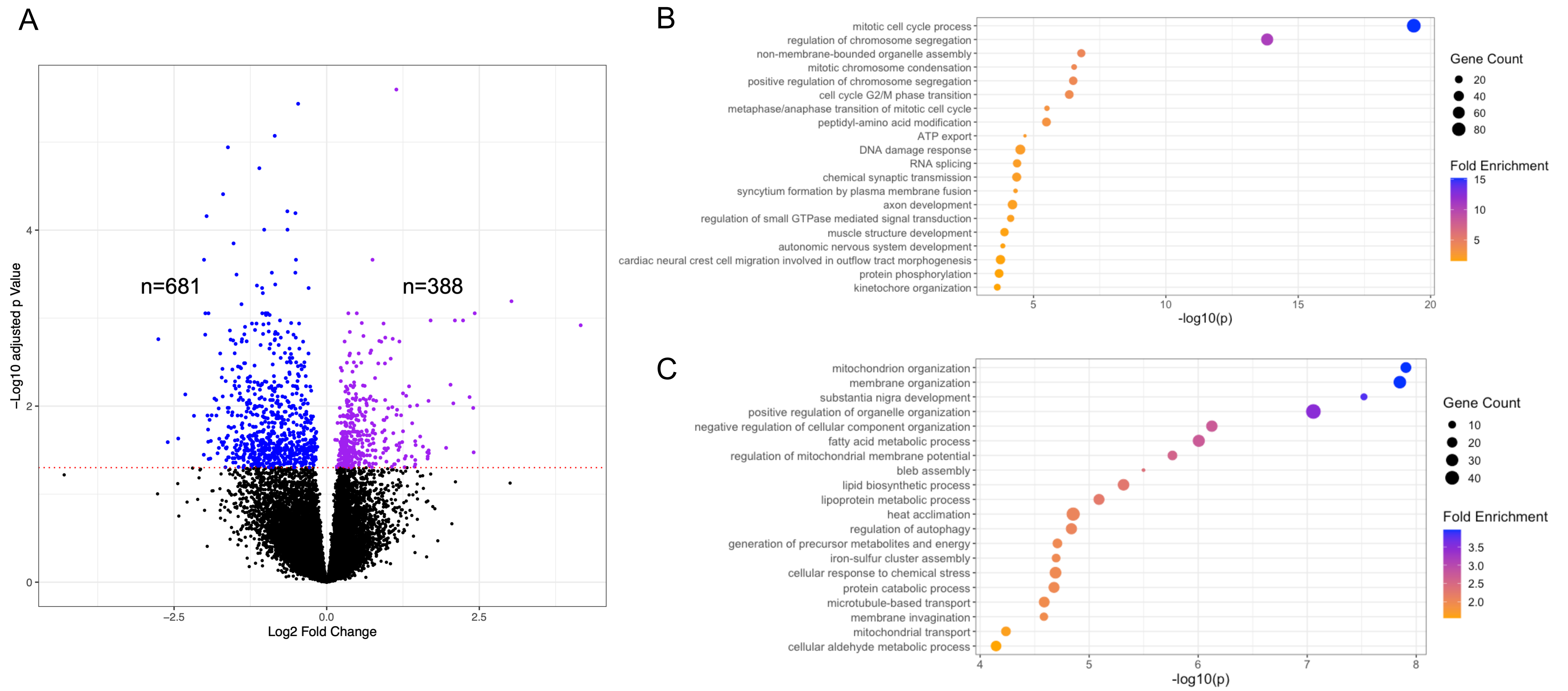

Supplementary Figure S6. Comparative RNA-seq analysis of cumulus cells collected from adolescents <16 years old and oocyte donors. A) Volcano plot shows downregulated (n=681) and upregulated DEGs (n=388) in adolescents (n=9) compared to donors (n=19) (dashed red line - adjusted p<0.05). B) Top 20 significantly downregulated GO terms (biological pathways) in adolescents <16 years old compared to donors. C) Top 20 significantly upregulated GO terms (biological pathways) in adolescents <16 years old compared to donors. DEGs: differentially expressed genes; GO: gene ontology.

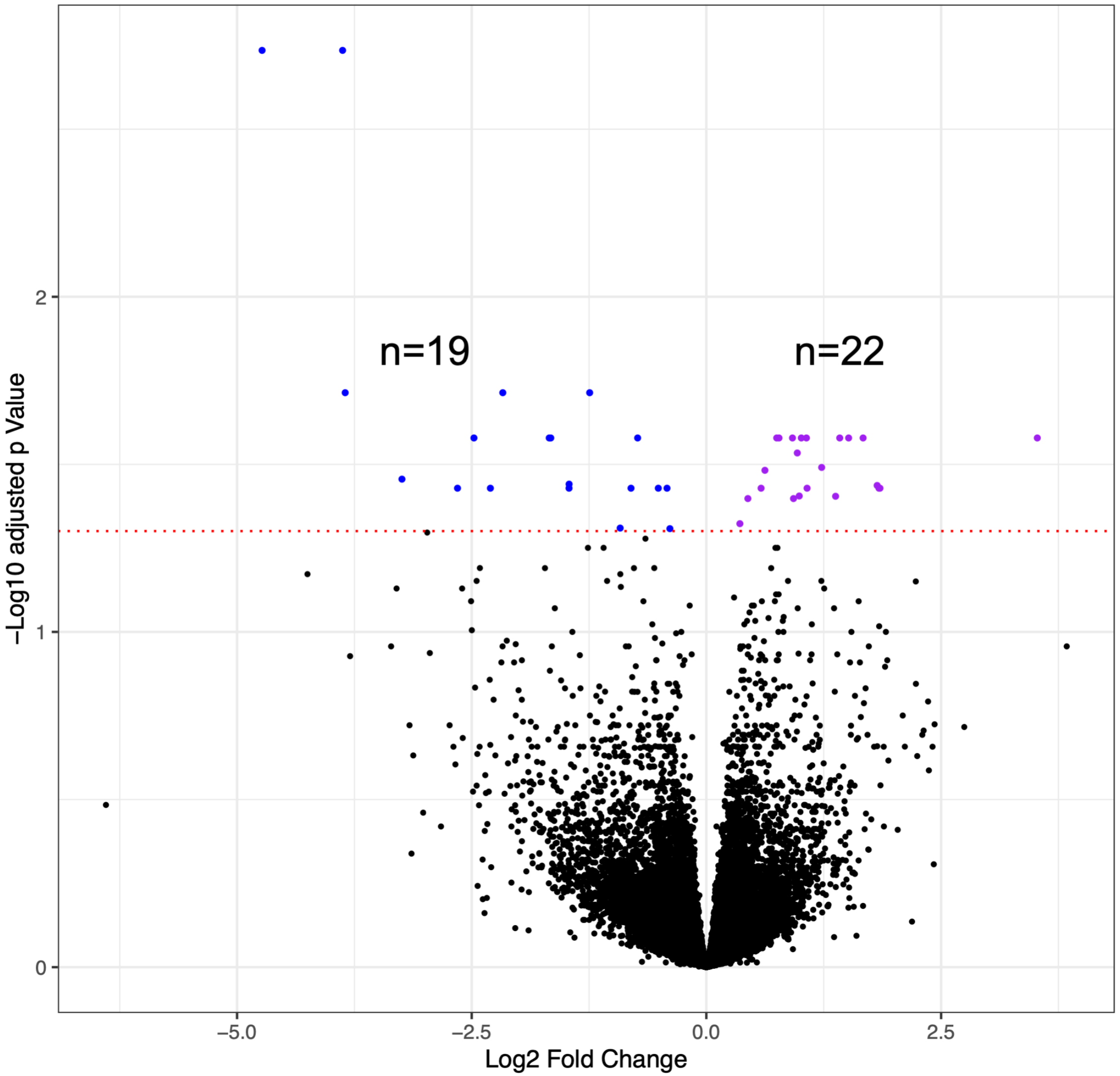

Supplementary Figure S7. Comparative RNA-seq analysis of cumulus cells collected from adolescents ≥16 and <16 years old. Volcano plot with downregulated (n=19) and upregulated DEGs (n=22) in adolescents ≥16 (n=9) compared to <16 years old (n=10) (dashed red line - adjusted p<0.05). DEGs: differentially expressed genes.

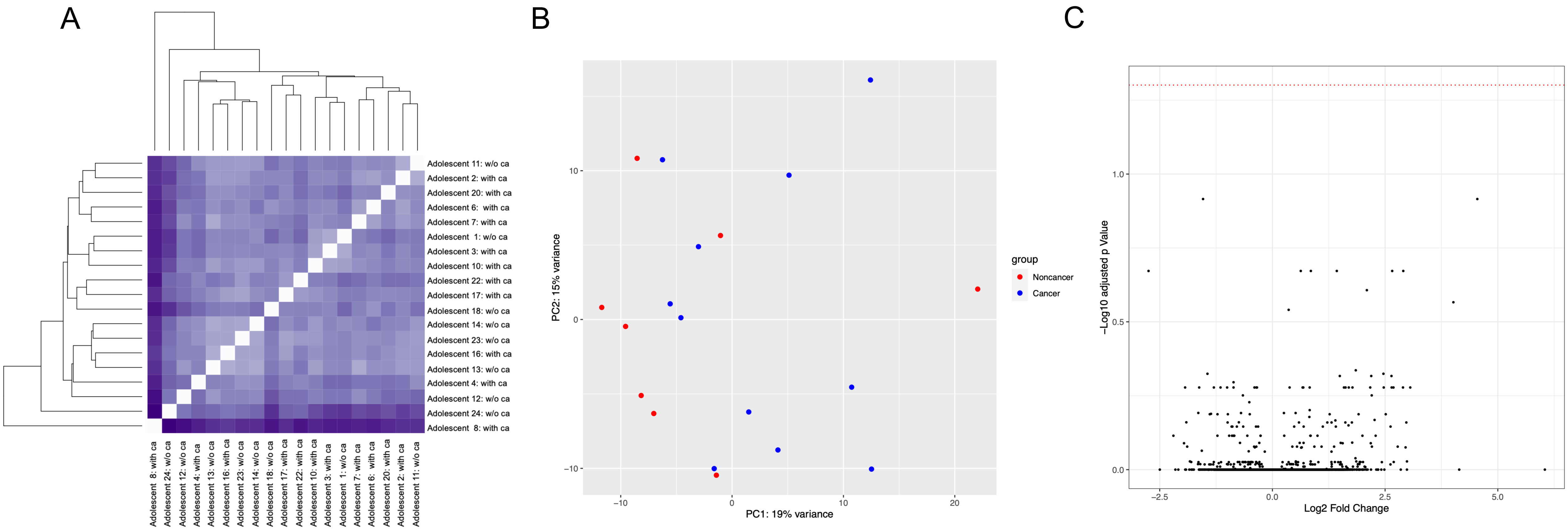

Supplementary Figure S8. RNA-seq analysis of cumulus cells collected from adolescents with cancer compared to adolescents without the cancer diagnosis. A) Unsupervised hierarchical clustering of adolescents with (n=11) and without (w/o) cancer (ca) (n=8) diagnosis B) Principal component analysis C) Volcano plot shows no DEGs between adolescents with and without cancer (dashed red line - adjusted p<0.05). DEGs: differentially expressed genes.

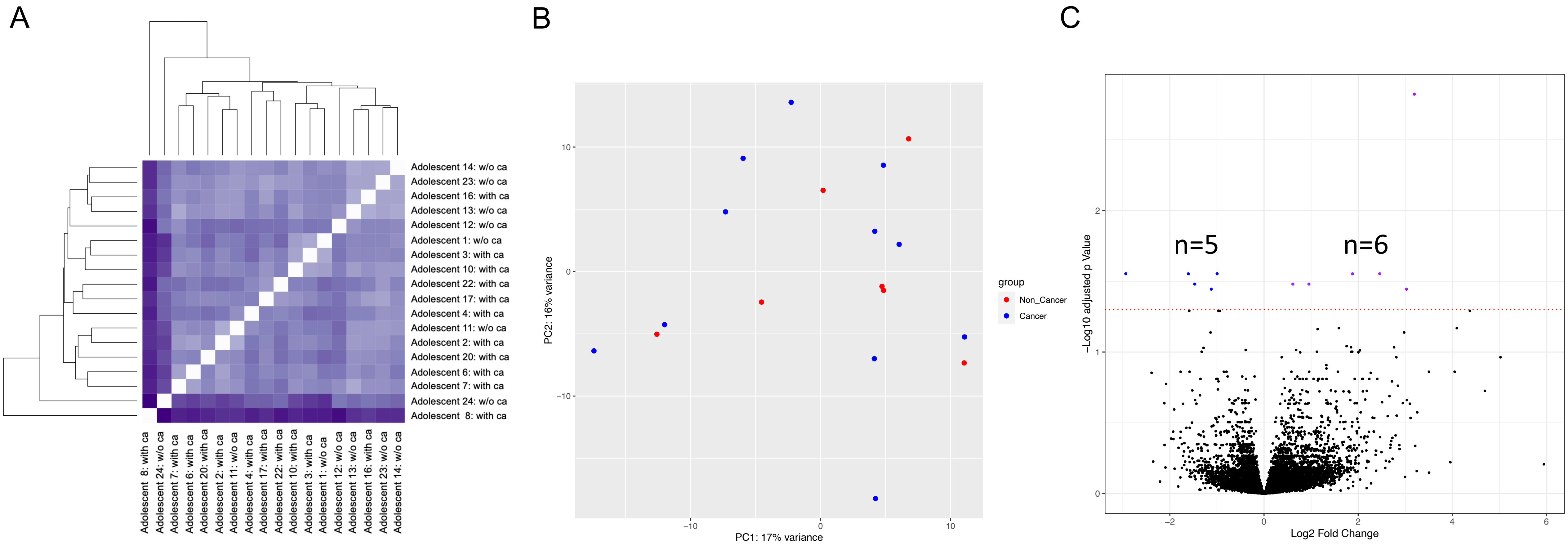

Supplementary Figure S9. RNA-seq analysis of cumulus cells collected from adolescents with cancer compared to adolescents without the cancer diagnosis after one possible outlier is removed from the initial data set. A) Unsupervised hierarchical clustering of adolescents with (n=11) and without (w/o) cancer diagnosis (ca) (n=7) B) Principal component analysis C) Volcano plot shows 11 DEGs between adolescents with and without cancer (dashed red line - adjusted p<0.05). DEGs: differentially expressed genes.

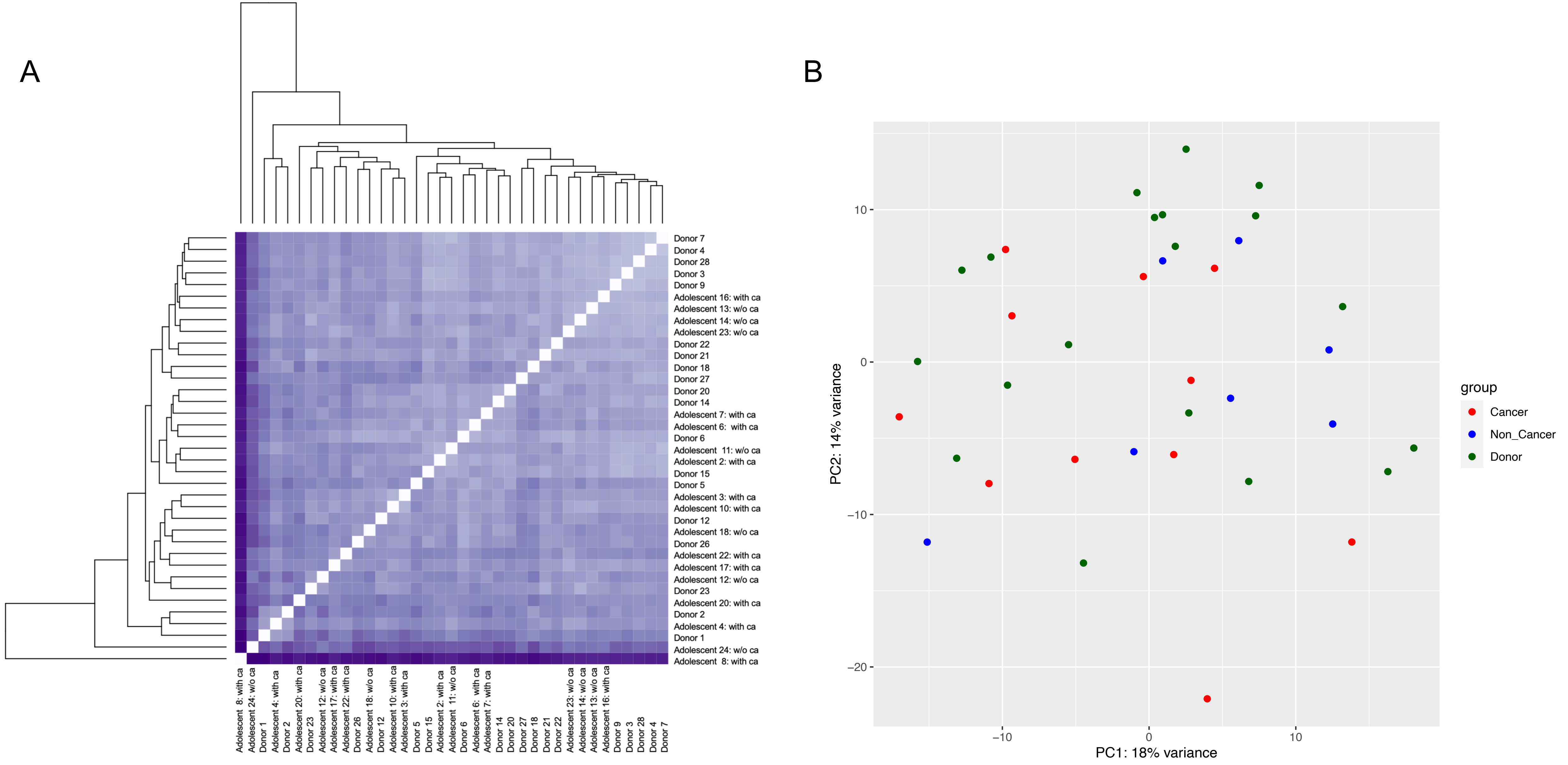

Supplementary Figure S10. RNA-seq analysis of cumulus cells collected from adolescents with (n=11) and without cancer (n=7; 10yo patient excluded) and oocyte donors (n=19). A) Unsupervised hierarchical clustering B) Principal component analysis

A

	Adolescents (n=18)	Donors (n=16)	P value
**Age** (years)	16.7 ± 0.6	27.3 ± 0.4	**<0.0001*** **
**BMI** (kg/m^2)	25.1 ± 1.7	23.6 ± 0.5	0.4032
**Race/ethnicity** (number of participants)			0.2785
Caucasian	9	14	
African American	3	1	
Asian	1	1	
Middle Eastern	1	0	
Caucasian-Hispanic	2	0	
**AMH** (ng/mL)	3.2 ± 0.5	5.8 ± 0.6	**0.0024*** **
**Antral follicle count (AFC)**	16.5 ± 1.2	23.3 ± 1.6	**0.0072*** **
**Luteal start**	44.44%	6.25%	**0.0189***
**Duration of stimulation** (days)	11.3 ± 0.3	11.4 ± 0.3	0.8799
**Number of monitoring visits** (days)	6.1 ± 0.3	6.8 ± 0.3	0.1373
**Type of USG**			**<0.0001*** **
Transvaginal%	38.89%	100.00%	
Transabdominal%	61.11%		
**Total gonadotropin dose** (IU)	5542 ± 471	4202 ± 435	0.0656
**Peak estradiol** (pg/mL)	2209 ± 234	3431 ± 303	**0.0031*** **
**Number of oocytes**	28.4 ± 3.4	29.9 ± 3.4	0.7506
**Number of MIIs**	19.4 ± 2.6	24.0 ± 2.9	0.1558
**Number of MIs**	1.6 ± 0.3	2.3 ± 0.4	0.2471
**Number of GVs**	3.3 ± 0.9	2.3 ± 0.6	0.4826
**Number of degenerated oocytes at retrieval**	1.5 ± 0.5	0.8 ± 0.3	0.1753
**Number of EZs**	2.1 ± 0.7	0.7 ± 0.4	**0.0394***

Values are presented as mean ± SEM. *P<0.05 and **P<0.01 is significant. BMI = body mass index; AMH = Anti-Mullerian hormone; MIIs = mature metaphase II arrested oocytes; MIs = immature oocytes between GV and MII stage. GVs = immature oocytes with germinal vesicle; EZs = zona pellucida devoid of an oocyte.

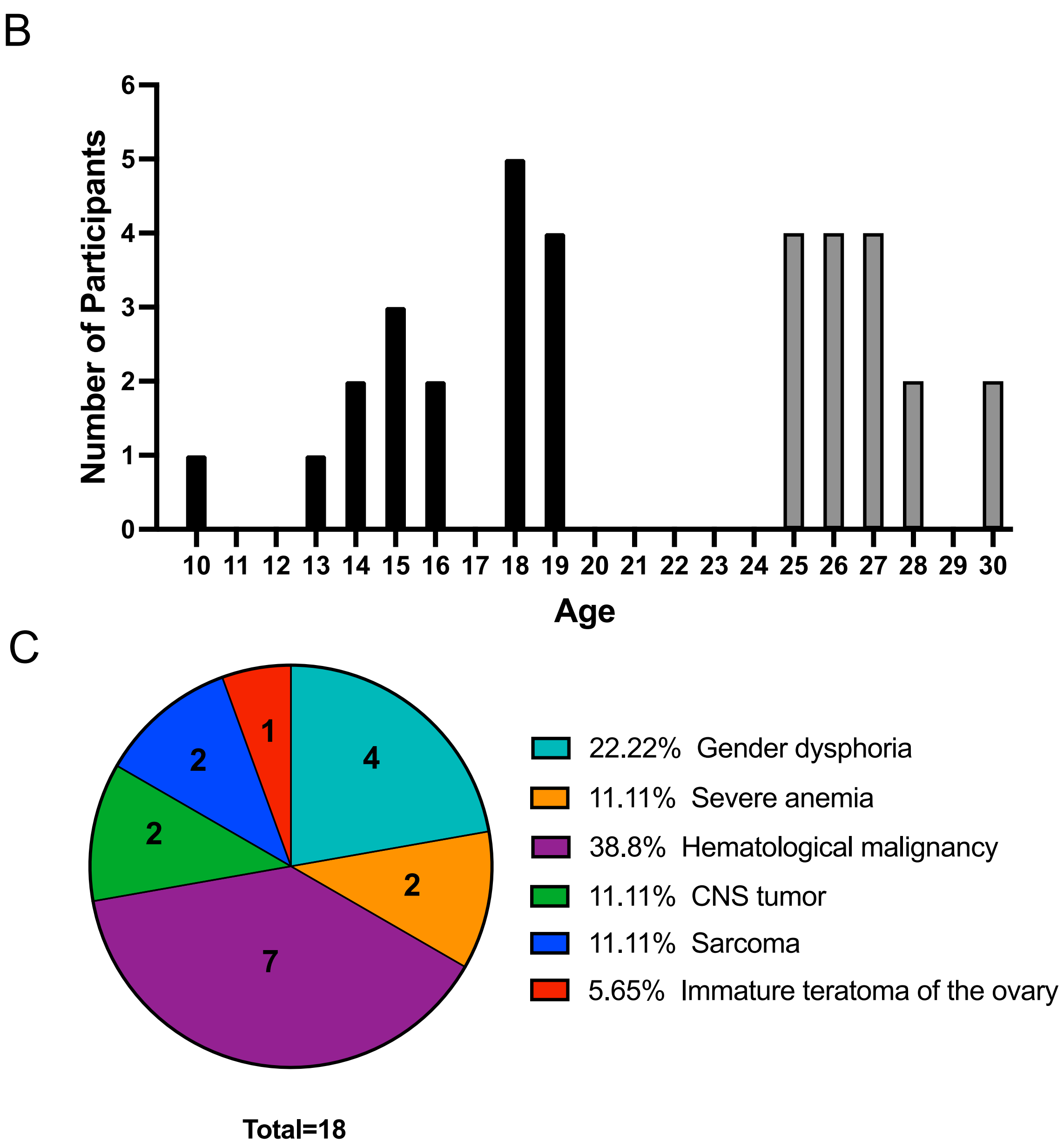

Supplementary Figure S11. Demographics and IVF cycle characteristics (A), age: adolescents (black bars), oocyte donors (grey bars) (B) of participants, and medical diagnoses of adolescents (C) included in follicular fluid cytokine analysis.

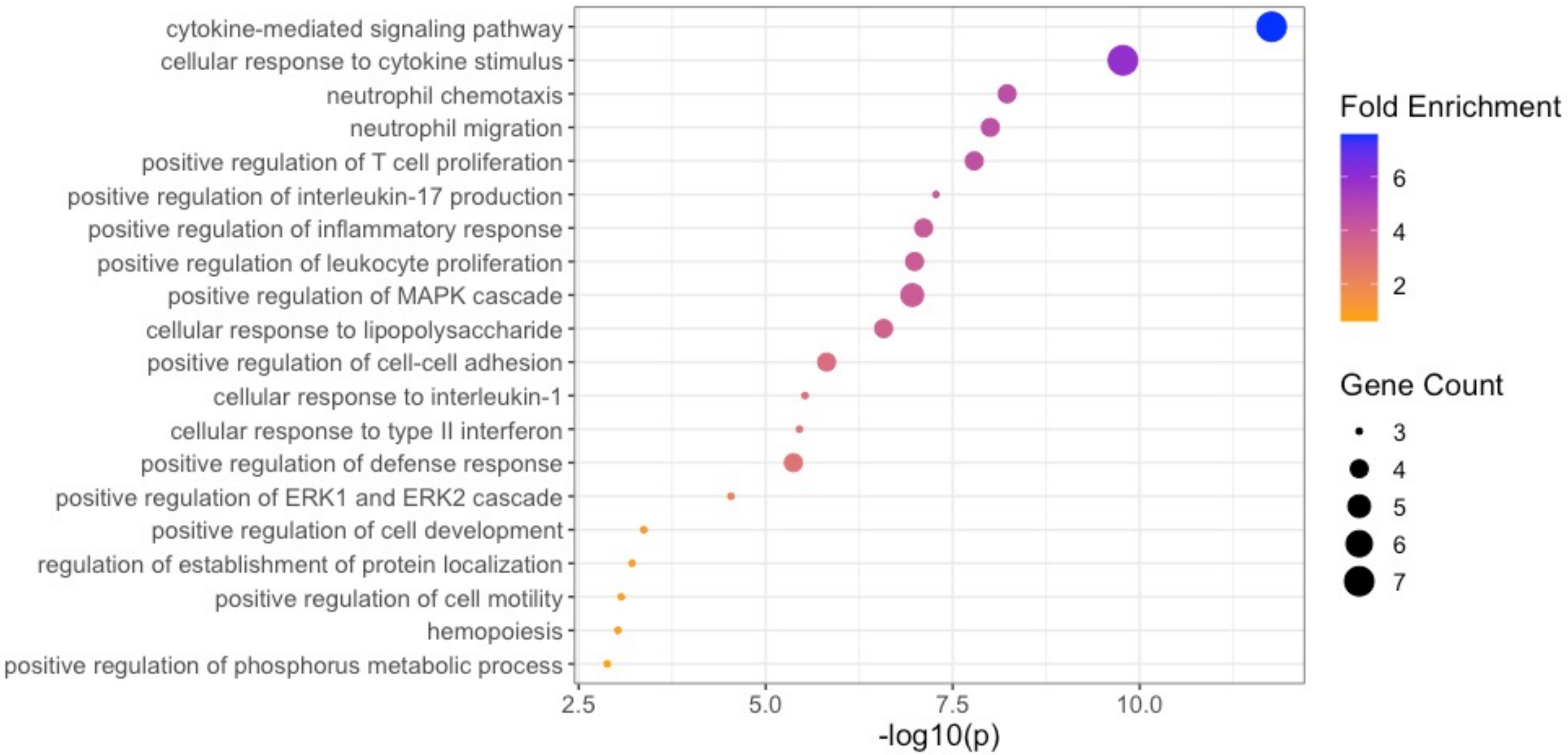

Supplementary Figure S12. Top 20 significantly different GO terms (biological pathways) based on follicular fluid (FF) cytokine levels in adolescents compared to oocyte donors. Enrichment analysis was performed based on significantly different FF cytokine levels (n=9) between adolescents and oocyte donors. GO: gene ontology.

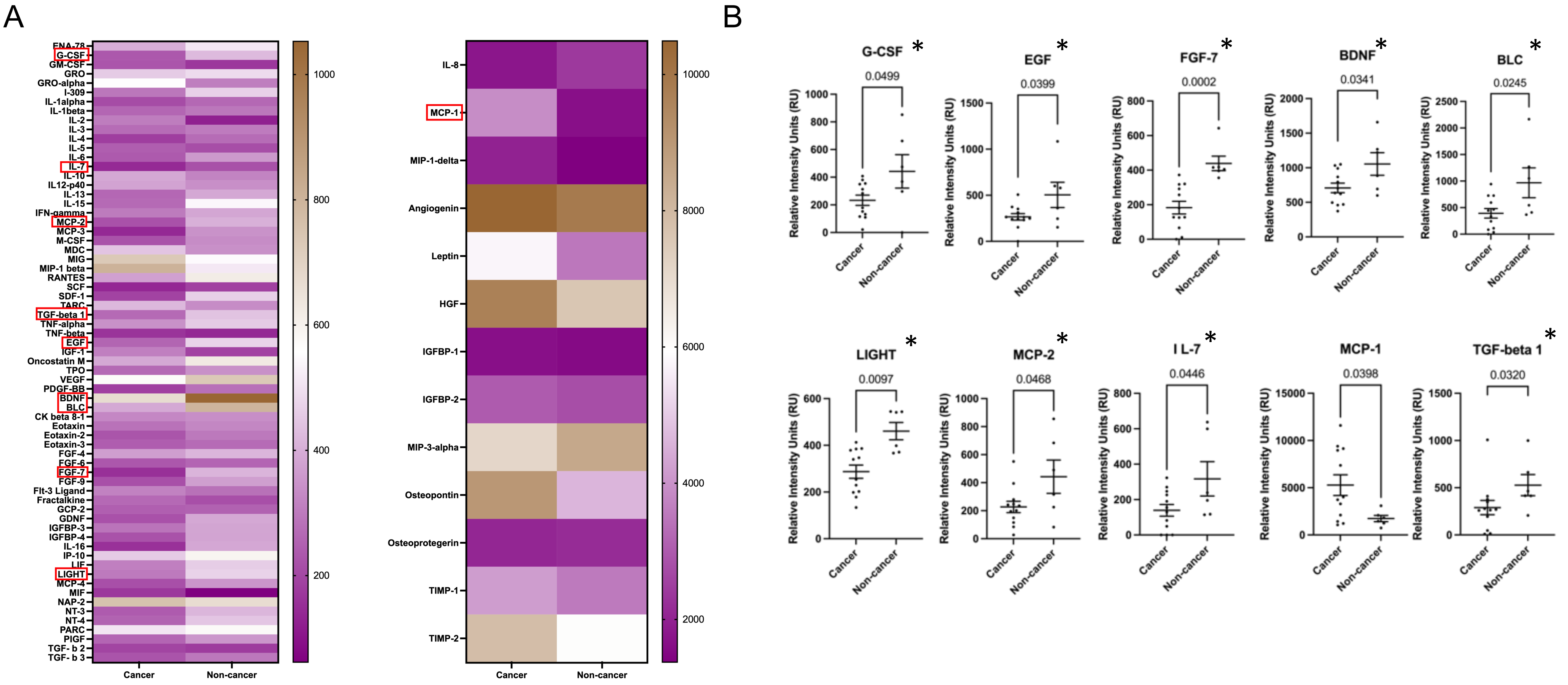

Supplementary Figure S13. The comparison of follicular fluid cytokine levels in adolescents with (n=12) and without cancer (n=6). A) Heatmap of follicular fluid levels of 80 cytokines in adolescents and oocyte donors with 10 of them significantly different between the two groups (red boxes) B) 9 out 10 cytokines demonstrated higher levels in non-cancer group (asterisks).

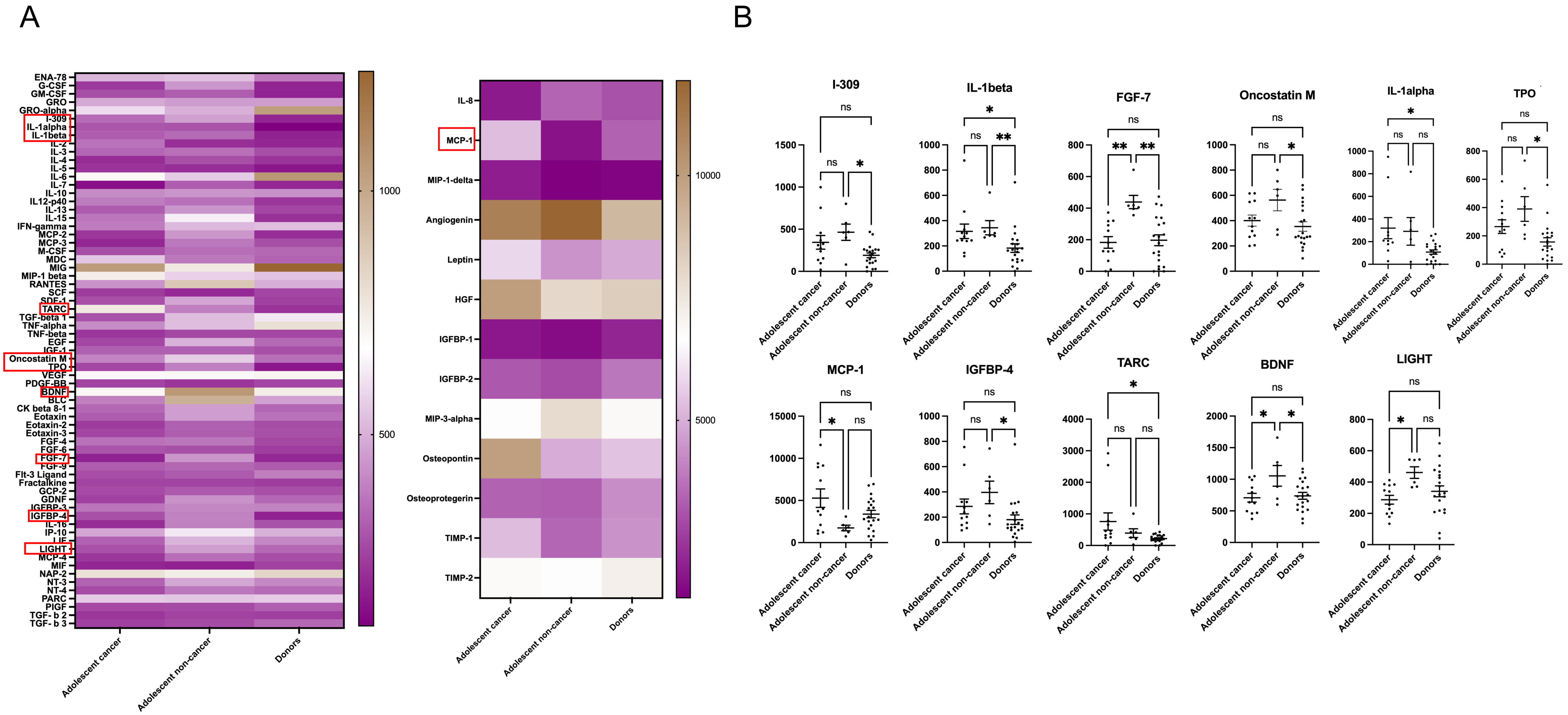

Supplementary Figure S14. The comparison of follicular fluid (FF) cytokine levels in oocyte donors (n=16) and adolescents with (n=12), and without cancer (n=6). A) Heatmap of FF levels of 80 cytokines in adolescents and oocyte donors with 11 of them significantly different across 3 groups (red boxes) B) Pairwise comparisons of FF levels of 11 cytokines across 3 groups (*p<0.05 and **p<0.01).
